# Novel insight into the aetiology of rheumatoid arthritis gained by a cross-tissue transcriptome-wide association study

**DOI:** 10.1136/rmdopen-2022-002529

**Published:** 2022-09-08

**Authors:** Jing Ni, Peng Wang, Kang-Jia Yin, Xiao-Ke Yang, Han Cen, Cong Sui, Guo-Cui Wu, Hai-Feng Pan

**Affiliations:** 1 Department of Epidemiology and Biostatistics, School of Public Health, Anhui Medical University, Hefei, Anhui, China; 2 Teaching Center for Preventive Medicine, School of Public Health, Anhui Medical University, Hefei, China; 3 Department of Rheumatology and Immunology, The First Affiliated Hospital of Anhui Medical University, Hefei, Anhui, China; 4 Department of Preventive Medicine, Ningbo University Medical School, Ningbo, Zhejiang, China; 5 Department of Orthopedics Trauma, The First Affiliated Hospital of Anhui Medical University, Hefei, Anhui, China; 6 Department of Obstetrics and Gynecological Nursing, School of Nursing, Anhui Medical University, Hefei, Anhui, China

**Keywords:** Rheumatoid Arthritis, Polymorphism, Genetic, Epidemiology

## Abstract

**Background:**

Although genome-wide association studies (GWASs) have identified more than 100 loci associated with rheumatoid arthritis (RA) susceptibility, the causal genes and biological mechanisms remain largely unknown.

**Methods:**

A cross-tissue transcriptome-wide association study (TWAS) using the unified test for molecular signaturestool was performed to integrate GWAS summary statistics from 58 284 individuals (14 361 RA cases and 43 923 controls) with gene-expression matrix in the Genotype-Tissue Expression project. Subsequently, a single tissue by using FUSION software was conducted to validate the significant associations. We also compared the TWAS with different gene-based methodologies, including Summary Data Based Mendelian Randomization (SMR) and Multimarker Analysis of Genomic Annotation (MAGMA). Further in silico analyses (conditional and joint analysis, differential expression analysis and gene-set enrichment analysis) were used to deepen our understanding of genetic architecture and comorbidity aetiology of RA.

**Results:**

We identified a total of 47 significant candidate genes for RA in both cross-tissue and single-tissue test after multiple testing correction, of which 40 TWAS-identified genes were verified by SMR or MAGMA. Among them, 13 genes were situated outside of previously reported significant loci by RA GWAS. Both TWAS-based and MAGMA-based enrichment analyses illustrated the shared genetic determinants among autoimmune thyroid disease, asthma, type I diabetes mellitus and RA.

**Conclusion:**

Our study unveils 13 new candidate genes whose predicted expression is associated with risk of RA, providing new insights into the underlying genetic architecture of RA.

**WHAT IS ALREADY KNOWN ON THIS TOPIC**

- Although genome-wide association studies (GWASs) have identified more than 100 rheumatoid arthritis (RA) risk loci, these variants can only explain 15% of RA heritability.
- Most GWAS signals are located in non-coding regions, where often overlap with gene regulatory elements and highly enrich expression quantitative trait loci.

**WHAT THIS STUDY ADDS**

- Through a two-stage transcriptome-wide association study design, we unveil 13 novel susceptibility genes whose genetically predicted expression is associated with risk of RA.
- Our study reveals shared genetic determinants among autoimmune thyroid disease, asthma, type I diabetes mellitus and RA.

**HOW THIS STUDY MIGHT AFFECT RESEARCH, PRACTICE OR POLICY**

- Our finding about novel RA loci refines the known genetic architecture of RA and provides genetic biomarkers for RA.

## Introduction

Rheumatoid arthritis (RA), a chronic autoimmune disease, is characterised by persistent synovial inflammation affecting around 0.5%–1.0% of general population worldwide.^1^ Currently, there is no completely curative therapy available for this highly disabling disease. Hence, a better knowledge of the underlying mechanisms of RA will contribute to the development of effective therapeutic targets.

Based on twin studies, the relative contribution of genetic variation to the liability of having RA has been estimated to be around 60%.^2^ Although genome-wide association studies (GWASs) have identified more than 100 RA risk loci, these variants can only explain 15% of RA heritability.^3^ In addition, the biological interpretation and functional comprehension of these associations remain elusive.^4 5^ Most GWAS signals are located in non-coding regions, where often overlap with gene regulatory elements and highly enrich expression quantitative trait loci (eQTL).^6 7^ These clues point to a crucial role of transcriptional regulation in impacting RA susceptibility.

Transcriptome-wide association study (TWAS) leverages eQTL and GWAS data to identify novel susceptibility genes whose genetically predicted expression is associated with disease risk.^8^ This method aggregates several variants into a functional gene unit, reducing the number of multiple comparisons and mapping to the candidate gene directly. As a powerful gene-based method, TWAS has been successfully applied to decipher the genetic architecture of several complex traits.^9–12^ The majority of existing TWAS studies calculated genetic–expression matrix in each tissue separately, which might neglect the sharing local regulation of gene expression across different tissues.^13^ Evidence has shown that eQTL with large effects can regulate gene expression across multiple tissues.^14^ A recent cross-tissue TWAS approach, called the unified test for molecular signatures (UTMOST), was developed to perform gene-level association analysis across different tissues, improving the accuracy and effectiveness of imputation model than that of single tissue method.^15^ By imposing a group-lasso penalty on the effect sizes of single nucleotide polymorphisms (SNPs) across tissues, UTMOST encourages eQTL that are shared across tissues and keeps tissue-specific eQTL with strong effects. In recent years, cross-tissue association analysis have been widely used in screening candidate susceptibility genes for complex multisystemic disorders whose biologically relevant tissues are not entirely clear, such as inflammatory bowel diseases, schizophrenia and Alzheimer’s disease.^16 17^

In this work, we carried out a cross-tissue TWAS of RA by integrating eQTL data from Genotype-Tissue Expression (GTEx) project with largest European RA GWAS. A single tissue TWAS using another Functional Summary-based Imputation (FUSION) method was adopted to validate candidate susceptibility genes synchronously. Follow-up bioinformatic analyses were performed to explore the biological characterisation for these candidate genes.

## Methods

### RA GWAS data source

We obtained the summary statistics from the largest meta-analysis of RA GWAS, comprising 14 361 cases and 43 923 controls of European ancestry.^18^ The participants enrolled in the GWAS meta-analysis were obtained from 18 separately studies. All patients were diagnosed with RA by professional rheumatologists according to the 1987 American College of Rheumatology criteria. Detailed description of the quality control, genotyping and imputation procedures were provided in the published studies.^19 20^

### TWAS analyses in cross-tissue and single tissue

**Figure 1:**
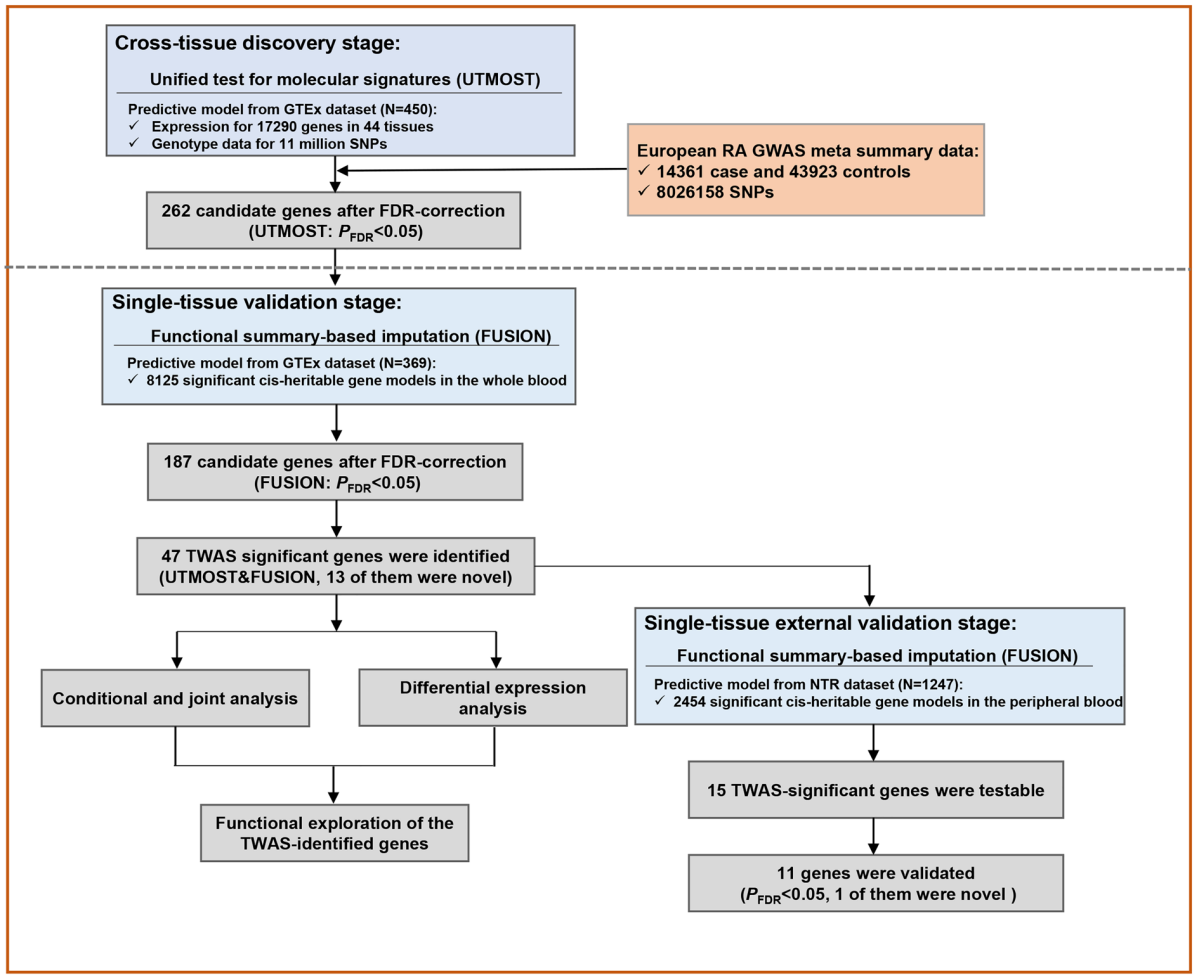
Overview of the transcriptome-wide association study design of rheumatoid arthritis (RA). FDR, false discovery rate; GWAS, genome-wide association study; TWAS, transcriptome-wide association study.

A two-stage TWAS design of the study is shown in figure 1. In the discovery stage, we performed a cross-tissue association test by using UTMOST method.^15^ RA GWAS summary data were integrated with eQTL data of 44 tissues from GTEx to impute the genetic component of gene expression in each tissue. Then, a generalised Berk-Jones test was applied to combine gene–trait associations in 44 GTEx human tissues based on the covariance from single tissue statistics (online supplemental table S1). For each gene, UTMOST trains a cross-tissue expression imputation model based on a penalised multivariate regression, which has considered the different directions and effect sizes of eQTL signals across tissues. To reduce noise in the cross-tissue association test and the risk of false positive rate, we performed a validation to incorporate RA GWAS and eQTL data of whole blood from GTEx by using the FUSION software,^8^ a widely used method in prior TWAS analysis. FUSION builds predictive models with several penalised linear models (GBLUP, LASSO, Elastic Net, etc) for those significant cis-heritability genes estimated by using SNPs within 500 kb on both sides of the gene boundary, and then chooses the best model based on the R^2^ calculated by a fivefold cross-validation of each model. In the external validation, we repeated the analysis using the eQTL data of peripheral blood from The Netherlands Twin Register (NTR)^21^ and calculated Pearson’s correlation coefficient to analyse the effect sizes of testable TWAS genes between GTEx and NTR. TWAS significance for both cross-tissue and single-tissue analysis was established as Benjamini-Hochberg corrected false discovery rate (FDR) value below 0.05.

10.1136/rmdopen-2022-002529.supp2 Supplementary data

### Conditional and joint analysis

Conditional and joint analysis for genome-wide FDR-corrected significant TWAS signals was used to evaluate the GWAS association signal after removing the TWAS association signal. Each RA GWAS SNP association was conditioned on the joint gene model. To investigate inflation of imputed association statistics under the null of no GWAS association, the permutation test was employed with a maximum of 100 000 permutations and permutation-based p value threshold of 0.05 for each of the significant loci.

### Gene-based association study

Summary data-based Mendelian randomization (SMR) is a novel gene-based approach to prioritise functional genes in GWAS loci, which uses cis-eQTL SNPs, gene expression and RA GWAS as instrumental variables, exposure and outcome, respectively.^22^ Multimarker analysis of genomic annotation (MAGMA) uses a multiple regression model to calculate the cumulative effect of several SNPs that assigned to a specific gene (±10 kb).^23^ The phase 3 of 1000 Genomes European population was used for reference panel to calculate the linkage disequilibrium (LD).^24^

### Validation of TWAS results by mRNA expression profiles of RA

To explore whether the expression levels of TWAS identified genes associated with RA risk were dysregulated in RA patients compared with controls, we obtained whole blood gene expression microarray data of RA from Gene Expression Omnibus dataset GSE93272.^25^ The genome-wide transcript profiling of whole blood was collected from 232 RA patients and 43 healthy controls. The gene expression data were processed with log transformation followed by quantile normalisation using the linear models for microarray data package to identify differentially expressed genes. We selected the differentially expressed genes based on FDR corrected p<0.05.

### Pathway enrichment analysis

To uncover the biologically relevant pathways in aetiology of RA, we conducted two gene-set methodologies including TWAS-based gene set enrichment analysis (TWAS-GSEA) and MAGMA-based gene-set analysis synchronously. TWAS-GSEA performs gene enrichment based on a mixed model test, in that it can directly use the results of TWAS. In MAGMA, the second gene-set analysis is established in a linear regression model by using the gene p value and gene correlation matrix. KEGG, BioCarta and Reactome pathways were downloaded from MSigDB database V.7.5.1 (https://www.gsea-msigdb.org/gsea/msigdb).

## Results

### Transcriptome-wide association study results of RA

For the cross-tissue discovery, a total of 262 genes showed a statistically significant signal after FDR correction (p<0.05) and 96 of them remained significant after Bonferroni correction (p<2.89×10^−6^) (online supplemental table S2) (online supplemental figure S1). For single-tissue internal validation, of all the 8125 genes modelled in our genotype data with significant cis-heritable expression in the whole blood from GTEx dataset, 187 genes displayed a significant TWAS association signal with *P*_FDR_<0.05 (online supplemental table S3). In all, we identified 47 overlapped candidate genes with the stringent threshold in both cross-tissue and single-tissue test, including 13 genes located in novel loci (table 1).

10.1136/rmdopen-2022-002529.supp1 Supplementary data

**Table 1:** The significant genes for RA risk in both cross-tissue and single-tissue TWAS analysis

Gene	CytoBand	BP0	BP1	UTMOST_Discovery		FUSION_Replication				Reported
				*P*_UTMOST_	*P*_FDR_	Top GWAS ID	Z score	*P*_FUSION_	*P*_FDR_	
*PTPN22*	1p13.2	114 356 433	114 414 381	1.82E-11	1.19E-08	rs2476601	6.75	1.51E-11	2.03E-09	Yes
*INPP5B*	1p34.3	38 326 369	38 412 729	1.24E-09	5.06E-07	rs28411352	4.98	6.29E-07	5.77E-05	Yes
*ATP13A2*	1p36.13	17 312 453	17 338 423	2.84E-04	2.13E-02	rs2240335	3.81	1.39E-04	8.44E-03	Yes
*MMEL1*	1p36.32	2 522 078	2 564 481	1.04E-08	3.57E-06	rs2843401	4.23	2.33E-05	1.61E-03	Yes
*FAM213B*	1p36.32	2 517 930	2 522 908	2.93E-09	1.14E-06	rs2843401	4.33	1.49E-05	1.08E-03	Yes
*TTC34*	1p36.32	2 567 415	2 718 286	3.86E-07	8.22E-05	rs2843401	−5.20	1.99E-07	1.91E-05	Yes
*FCRL3*	1q23.1	157 644 111	157 670 647	3.47E-06	5.31E-04	rs3761959	4.38	1.20E-05	8.89E-04	Yes
*PUS10*	2p15	61 167 357	61 245 394	2.55E-11	1.38E-08	rs34695944	5.31	1.10E-07	1.11E-05	Yes
*AFF3*	2q11.2	100 162 323	100 759 201	2.92E-07	6.51E-05	rs9653442	5.56	2.67E-08	2.80E-06	Yes
*PSD4*	2q14.1	113 914 902	113 966 973	2.42E-04	1.87E-02	rs3791336	−3.55	3.85E-04	2.03E-02	No
*PDHB*	3p14.3	58 413 357	58 419 584	3.03E-04	2.22E-02	rs2176082	−3.50	4.71E-04	2.39E-02	Yes
*FBXO40*	3q13.33	121 311 966	121 349 139	7.05E-04	4.27E-02	rs4413346	3.29	1.01E-03	4.46E-02	No
*DAP*	5p15.2	10 679 342	10 761 384	9.19E-05	8.65E-03	rs267949	−3.32	8.93E-04	4.05E-02	No
*PPIP5K2*	5q21.1	102 455 853	102 548 500	3.99E-05	4.34E-03	rs35801	3.47	5.28E-04	2.61E-02	Yes
*PAM*	5q21.1	102 089 685	102 366 809	7.33E-09	2.64E-06	rs26258	−3.35	8.11E-04	3.70E-02	Yes
*IP6K3*	6p21.31	33 689 444	33 714 762	4.42E-10	1.91E-07	rs2296330	6.94	3.89E-12	5.51E-10	Yes
*C6orf106*	6p21.31	34 555 065	34 664 636	2.14E-06	3.49E-04	rs2814943	3.26	1.10E-03	4.80E-02	Yes
*PGBD1*	6p22.1	28 249 314	28 270 326	3.19E-05	3.55E-03	rs2232428	−6.47	1.00E-10	1.26E-08	No
*TRIM10*	6p22.1	30 119 722	30 128 711	7.28E-12	6.12E-09	rs1362126	4.38	1.20E-05	8.89E-04	No
*TRIM27*	6p22.1	28 870 779	28 891 766	1.52E-13	4.60E-10	rs13190937	−4.12	3.79E-05	2.51E-03	No
*ZNF322*	6p22.2	26 636 518	26 659 980	7.11E-05	7.08E-03	rs17539358	3.74	1.85E-04	1.07E-02	No
*HIST1H2BG*	6p22.2	26 216 428	26 216 872	4.00E-04	2.76E-02	rs17539358	−3.31	9.20E-04	4.15E-02	No
*IRF5*	7q32.1	128 577 666	128 590 089	6.43E-13	1.22E-09	rs10488631	5.67	1.40E-08	1.55E-06	Yes
*FAM167A*	8p23.1	11 278 972	11 332 224	8.38E-05	8.03E-03	rs13277113	4.68	2.88E-06	2.40E-04	Yes
*TRAF1*	9q33.2	123 664 671	123 691 451	3.95E-05	4.34E-03	rs10818482	4.26	2.07E-05	1.47E-03	Yes
*PSMD5*	9q33.2	123 577 774	123 605 262	1.30E-04	1.16E-02	rs10818482	3.75	1.75E-04	1.02E-02	Yes
*PHF19*	9q33.2	123 617 977	123 639 606	9.31E-08	2.35E-05	rs10818482	−5.14	2.80E-07	2.63E-05	Yes
*ZNF438*	10p11.23	31 109 136	31 320 866	7.83E-05	7.65E-03	rs867768	−3.50	4.58E-04	2.36E-02	Yes
*TMEM258*	11q12.2	61 535 973	61 560 274	4.94E-04	3.24E-02	rs968567	−4.77	1.87E-06	1.59E-04	Yes
*FADS1*	11q12.2	61 567 099	61 596 790	1.08E-04	9.77E-03	rs968567	−4.55	5.30E-06	4.20E-04	Yes
*DDX6*	11q23.3	118 620 034	118 661 858	2.01E-07	4.62E-05	rs4938573	3.80	1.43E-04	8.62E-03	Yes
*YAF2*	12q12	42 550 906	42 632 151	6.10E-04	3.82E-02	rs7954523	3.30	9.70E-04	4.35E-02	No
*SUOX*	12q13.2	56 390 964	56 400 425	1.10E-07	2.70E-05	rs705699	5.39	7.02E-08	7.18E-06	Yes
*RPS26*	12q13.2	56 435 637	56 438 116	8.40E-08	2.18E-05	rs705699	−5.06	4.17E-07	3.87E-05	Yes
*METTL21B*	12q14.1	58 165 275	58 176 324	1.03E-05	1.34E-03	rs238516	−4.40	1.06E-05	8.08E-04	Yes
*TSPAN31*	12q14.1	58 131 796	58 143 994	3.70E-06	5.50E-04	rs238516	4.43	9.60E-06	7.38E-04	Yes
*RNF40*	16p11.2	30 773 066	30 787 628	6.05E-06	8.28E-04	rs8058578	3.86	1.13E-04	6.91E-03	No
*SPNS1*	16p11.2	28 985 542	28 995 869	1.57E-04	1.34E-02	rs7500321	4.15	3.25E-05	2.21E-03	No
*PGAP3*	17q12	37 827 375	37 853 050	7.72E-07	1.52E-04	rs2872507	−4.63	3.73E-06	3.01E-04	Yes
*GSDMB*	17q21.1	38 060 848	38 076 107	6.70E-09	2.47E-06	rs2872507	−5.44	5.34E-08	5.53E-06	Yes
*IKZF3*	17q21.1	37 921 198	38 020 441	5.59E-06	7.76E-04	rs2872507	5.58	2.34E-08	2.49E-06	Yes
*ORMDL3*	17q21.1	38 077 294	38 083 854	5.87E-08	1.64E-05	rs2872507	−5.65	1.64E-08	1.79E-06	Yes
*TSSK6*	19p13.11	19 623 227	19 626 838	4.95E-07	1.04E-04	rs11085264	3.76	1.70E-04	1.00E-02	No
*TYK2*	19p13.2	10 461 209	10 491 352	8.01E-07	1.55E-04	rs2304256	−6.92	4.46E-12	6.21E-10	Yes
*OPRL1*	20q13.33	62 711 526	62 731 996	5.97E-04	3.75E-02	rs4408777	−3.71	2.10E-04	1.18E-02	No
*TMEM50B*	21q22.11	34 804 792	34 852 318	3.06E-04	2.23E-02	rs11702844	4.12	3.76E-05	2.51E-03	Yes
*UBASH3A*	21q22.3	43 824 008	43 867 791	2.49E-08	7.87E-06	rs1893592	−5.73	9.81E-09	1.10E-06	Yes

BP0, start base position; BP1, end base position, hg19/GRCh37 build; CytoBand, cytogenic band where the variant is positioned Top GWAS ID, rsID of the most significant GWAS SNP in locus; Reported, reported genes implicated in previous GWASs.

FUSION, Functional Summary-based Imputation; GWAS, genome-wide association study; RA, rheumatoid arthritis; TWAS, transcriptome-wide association study; UTMOST, unified test for molecular signatures.

### Conditional and joint analysis

**Figure 2:**
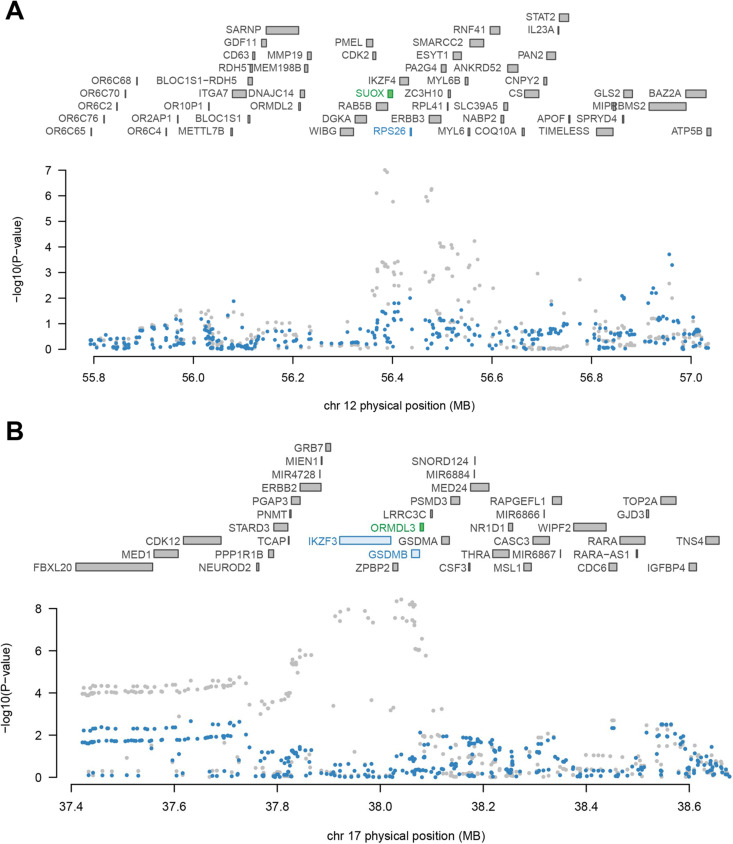
Regional association of transcriptome-wide association study (TWAS) hits. (A) Chromosome 12q13.2 regional association plot. (B) Chromosome 17q21.1 regional association plot. The top panel highlights all genes in the region. The marginally associated TWAS genes are shown in blue, and the jointly significant genes are shown in green. The bottom panel show a regional Manhattan plot of the genome-wide association study (GWAS) data before (grey) and after (blue) conditioning on the predicted expression of the green genes.

As shown in table 2, 11 loci represented the independent signal containing multiple significant genes, including 1p36.32 (*TTC34*), 5q21.1 (*PPIP5K2*), 6p21.31 (*IP6K3*, *C6orf106*), 6p22.1 (*PGBD1, TRIM10*), 6p22.2 (*HIST1H2BG*, *ZNF322*), 9q33.2 (*PHF19*, *TRAF1*), 11q12.2 (*TMEM258*), 12q13.2 (*SUOX*), 12q14.1 (*TSPAN31*), 16p11.2 (*SPNS1*, *RNF40*) and 17q21.1 (*ORMDL3*) (conditional p<0.05). We observed that several GWAS signals were driven by genetically modulated gene expression. For instance, *SUOX* explained most of the signal at 12q13.2 locus, while TWAS signal for *RPS26* dropped obviously when conditioned on the predicted expression of *SUOX* (figure 2A). Similarly, a cluster of three TWAS significant associations (*ORMDL3, GSDMB* and *PGAP3*) were found at 17q21.1 locus, conditional analysis indicated that *ORMDL3* explained most of the signals in this region (figure 2B).

**Table 2:** Conditional analysis result of multigene loci

CytoBand	Genes in locus	Gene	TWAS.Z	TWAS.P	Cond.Z	Cond.P
1p36.32	*TTC34, MMEL1, FAM213B*	***TTC34***	−5.20	2.00E-07	−5.20	2.00E-07
		*FAM213B*	4.30	1.50E-05	−0.07	9.50E-01
		*MMEL1*	4.20	2.30E-05	−0.07	9.50E-01
5q21.1	*PPIP5K2, PAM*	***PPIP5K2***	3.50	5.30E-04	3.50	5.30E-04
		*PAM*	−3.30	8.10E-04	−1.10	2.50E-01
6p21.31	*IP6K3, C6orf106*	***IP6K3***	6.90	3.90E-12	6.90	3.90E-12
		***C6orf106***	3.30	1.10E-03	3.30	1.10E-03
6p22.1	*PGBD1, TRIM10, TRIM27*	***PGBD1***	−6.50	1.00E-10	−6.50	1.00E-10
		***TRIM10***	4.40	1.20E-05	2.35	1.90E-02
		*TRIM27*	−4.10	3.80E-05	0.04	9.68E-01
6p22.2	*HIST1H2BG, ZNF322*	***HIST1H2BG***	−3.30	9.20E-04	−2.30	2.08E-02
		***ZNF322***	3.70	1.80E-04	2.90	3.90E-03
9q33.2	*PHF19, TRAF1, PSMD5*	***PHF19***	−5.10	2.80E-07	−3.70	1.80E-04
		***TRAF1***	4.30	2.10E-05	2.40	1.62E-02
		*PSMD5*	3.80	1.70E-04	−0.24	8.10E-01
11q12.2	*TMEM258, FADS1*	***TMEM258***	−4.80	1.90E-06	−4.80	1.90E-06
		*FADS1*	−4.60	5.30E-06	0.07	9.50E-01
12q13.2	*SUOX, RPS26*	***SUOX***	5.40	7.00E-08	5.40	7.00E-08
		*RPS26*	−5.10	4.20E-07	−0.13	9.00E-01
12q14.1	*TSPAN31, METTL21B*	***TSPAN31***	4.40	9.60E-06	4.40	9.60E-06
		*METTL21B*	−4.40	1.10E-05	−1.30	1.80E-01
16p11.2	*SPNS1, RNF40*	***SPNS1***	4.20	3.20E-05	4.20	3.30E-05
		***RNF40***	3.90	1.10E-04	3.90	1.10E-04
17q21.1	*ORMDL3, IKZF3, GSDMB*	***ORMDL3***	−5.60	1.60E-08	−5.60	1.60E-08
		*IKZF3*	5.60	2.30E-08	0.76	4.50E-01
		*GSDMB*	−5.40	5.30E-08	0.14	8.90E-01

*Bold gene was the independent Transcriptome-wide association study signal in the locus.

Cond.P, p values of the gene after conditional analysis; Cond.Z, Z-score of the gene after conditional analysis; FUSION, Functional Summary-based Imputation; TWAS, transcriptome-wide association study; TWAS.P, p values of the gene in FUSION TWAS analysis; TWAS.Z, Z-score of the gene in FUSION TWAS analysis.

### Integrating peripheral blood eQTL for external validation

Compared with the whole blood eQTL dataset from GTEx, peripheral blood eQTL yielded fewer testable genes (n=2454) (online supplemental table S4). Across all common testable genes (n=1686), the effect sizes of those genes between GTEx and NTR were highly correlated (R=0.60, p<0.001) (online supplemental figure S2). Among the 47 TWAS significant genes, there were 15 testable genes in the replication stage. Finally, TWAS analysis using the independent peripheral blood eQTL data successfully validated 11 genes as RA risk genes (online supplemental table S5).

### Comparison of TWAS with different gene-based methodologies

Venn plots illustrated the number of nominal significant genes achieved by the four methodologies. The cross-tissues test achieved more significantly associated genes, showing 67.6%, 148.7% improvement compared with FUSION and SMR, respectively (online supplemental figure S3A). Consistently, such improvement was observed within FDR-corrected thresholds. Among the 47 identified TWAS-significant associations, 85.1% (40/47) were recovered by SMR or MAGMA after FDR correction (online supplemental figure S3B).

**Figure 3:**
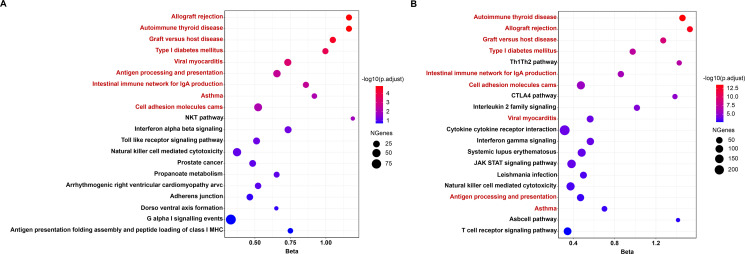
The top 20 most significant gene set enrichment results based on (A) TWAS-based gene set enrichment analysis (TWAS-GSEA) (B) MAGMA-based gene set enrichment analysis. nine gene-sets shared between TWAS-GSEA and MAGMA-based gene set enrichment analysis with FDR <0.05 were marked in red. FDR, false discovery rate; MAGMA, multimarker analysis of genomic annotation; TWAS, transcriptome-wide association study.

### Differentiation analysis in RA and controls

To explore whether these 47 risk genes identified in TWAS analysis are differentially expressed between RA patients and health controls, we evaluated their mRNA expression levels in whole blood from RA patients and healthy controls. In lines with TWAS results, eight genes including *PTPN22*, *PUS10*, *PPIP5K2*, *ZNF322*, *IRF5*, *PSMD5*, *YAF2* and *TMEM50B* were remarkably overexpressed, while seven genes including *PGBD1, PHF19, PGAP3, GSDMB, ORMDL3, TYK2* and *UBASH3A* were decreased in RA patients compared with healthy controls (online supplemental table S6).

### Pathway enrichment analysis

Across all the 828 gene-sets with at least 10 cis-heritable genes, 10 gene-sets were significantly enriched after multiple testing correction (online supplemental table S7). MAGMA-based gene-set analysis suggested that 45 gene-sets were significantly enriched (*P*_FDR_<0.05) (online supplemental table S8). Among the top 20 enriched pathways, nine significant gene-sets were consistent in both TWAS-GSEA and MAGMA (figure 3). The results provided strong evidence of shared aetiology among autoimmune thyroid disease, asthma, type I diabetes and RA and pointed out several crucial immune-related signalling such as the Th1/Th2, intestinal immune network for IGA production, antigen processing and presentation in the pathogenesis of RA.

## Discussion

With the currently released largest RA GWAS dataset, we systematically estimated the associations between genetically predicted gene expressions and RA risk. A total of 47 susceptibility genes for RA were prioritised through a two-stage TWAS design, and 13 of which resided in the novel loci. Together with in silico analysis deepened our understanding of genetic architecture and comorbidity aetiology of RA.

According to our results, the cross-tissue TWAS approach can effectively achieve more significantly associated genes compared with other two single-tissue gene-based methodologies. A recent cross-tissue TWAS has discovered two novel carcinogenic susceptibility genes for lung cancer, of which the design is similar to that of our study.^26^ Liu *et al*^27^ conducted a cross-tissue TWAS for pancreatic cancer, in which they revealed 13 significant gene-level associations at an FDR below 0.05, including six new susceptibility genes. Focused on RA, there has been a study of TWAS that generates genetic expression matrix in four tissues from GTEx dataset separately with FUSION software.^28^ The authors reported a total of 692 significant TWAS genes with p value less than 0.05. To circumvent the drawbacks of single-tissue analysis, we adopted cross-tissue analyses for discovery strategy in order to identify more reliable genes. Additionally, with expanded data from GTEx project and a more strict threshold of significance, our study is likely to achieve stable and accurate results.

Several TWAS-significant genes with strong evidence from previous functional studies were located at the known RA loci. For example, hyperactivity of *PTPN22* in vitro might lead to produce reactive oxygen species, driving RA through abnormal inflammatory response and joint damage.^29^ *PTPN22* has also been suggested as a preclinical molecular signature for RA.^30^ Existing evidence has proved that a part of novel RA genes exhibits immune-related features. For instance, *ZNF322* at 6p22.2, which belongs to the zinc finger protein family, has been reported to act as a transcriptional activator in MAPK signalling pathways.^31^ Apart from this, *ZNF322* can regulate the expression of two cell-cycle genes (*P27* and *CDK2*), which were characterised as crucial regulators involved in synoviocytes proliferation.^32–35^ *SPNS1,* which encodes Spinster homologue 1 protein, is a transmembrane protein that can modulate the metabolism of autophagic lysosomal and critically associate with cellular ageing and survival.^36 37^ Notably, *SPNS1* has recently been considered as a core component of T cell receptor signalling.^38^ A slew of studies have shown that *TRIM10* and *TRIM27* were involved in several inflammation processes.^39–41^ TRIM10 exhibited a lower expression in patients with systemic lupus erythematosus than in healthy individuals, which negatively induced the IFN/JAK/STAT signalling pathway through impacting the interaction between IFNAR1 and TYK2.^39^ Of note is that *TRIM10* has been reported to be associated with systemic juvenile idiopathic arthritis.^42^ Moreover, Liu *et al*^43^ demonstrated that *TRIM27* modulated the proliferation of mesangial cell in kidneys of lupus nephritis. It has been suggested that knockdown of *TRIM27* inhibited the endothelial cells injuries in lupus nephritis via the FoxO1 signalling pathway.^44^

Numerous observational studies have observed the coexistence of RA and other immune-mediated diseases, consistent with significant genetic correlations between them.^45–48^ In our work, both TWAS-based and MAGMA-based enrichment analysis also illustrated the shared genetic determinants between autoimmune thyroid disease, asthma, type I diabetes mellitus and RA. Speculatively, there are common immune regulatory mechanisms among these diseases. Insights gained from these results will provide further therapeutic directions for immune-mediated diseases.

Although TWAS has advantages in improving statistical power and avoiding reverse causality, some caveats should be noted in this study. First, not all genes can be captured due to the criterion of significant cis-heritability genes in the TWAS analyses, and those SNPs influencing RA but are independent of cis expression will be ignored. Second, the genetically predicted models were measured in multiple tissues but not in the biologically relevant tissues such as synovial tissues and immune cells. As high-throughput data continue to be released for more cell types and tissues, as well as a larger RA GWAS dataset of 276 020 samples from five ancestral groups,^49^ cross-tissue association analysis promises to show even better effectiveness and provides greater insights for RA genetics. Finally, the underlying mechanisms of association signals have not been validated through experimental techniques.

In summary, we reveals 13 novel susceptibility genes whose genetically predicted expression is associated with risk of RA, providing new insights into the underlying genetic architecture of RA. However, further functional studies are still needed to elucidate the underlying biological activities of these significant signals.

## Data Availability

The datasets used and/or analysed during the current study are available from the corresponding authors on reasonable request.
