# Supplementary material for: Novel insight into the aetiology of rheumatoid arthritis gained by a cross-tissue transcriptome-wide association study
Source: RMD Open. 2022 Sep 8;8(2):e002529. doi: 10.1136/rmdopen-2022-002529 (PMC9462377; doi:10.1136/rmdopen-2022-002529)
Supplement: Supplementary data [file rmdopen-2022-002529supp002.pdf]

Supplementary Table 1. Sample sizes of 44 GTEx v6p tissues used in UTMOST

Tissue	Sample size
Adipose - Subcutaneous	298
Adipose - Visceral Omentum	185
Adrenal Gland	126
Artery - Aorta	197
Artery - Coronary	118
Artery - Tibial	285
Brain - Anterior cingulate cortex (BA24)	72
Brain - Caudate (basal ganglia)	100
Brain - Cerebellar Hemisphere	89
Brain - Cerebellum	103
Brain - Cortex	96
Brain - Frontal Cortex (BA9)	92
Brain - Hippocampus	81
Brain - Hypothalamus	81
Brain - Nucleus accumbens (basal ganglia)	93
Brain - Putamen (basal ganglia)	82
Breast - Mammary Tissue	183
Cells - EBV-transformed lymphocytes	114
Cells - Transformed fibroblasts	272
Colon - Sigmoid	124
Colon - Transverse	169
Esophagus - Gastroesophageal Junction	127
Esophagus - Mucosa	241
Esophagus - Muscularis	218
Heart - Atrial Appendage	159
Heart - Left Ventricle	190
Liver	97
Lung	278
Muscle - Skeletal	361
Nerve - Tibial	256
Ovary	85
Pancreas	149
Pituitary	87
Prostate	87
Skin - Not Sun Exposed (Suprapubic)	196
Skin - Sun Exposed (Lower leg)	302
Small Intestine - Terminal Ileum	77
Spleen	89
Stomach	170
Testis	157
Thyroid	278
Uterus	70

Vagina	79
Whole Blood	338

Supplementary Table 2. TWAS summary statistics in cross-tissue test using 44 GTEx tissues (P_{FDR}<0.05)

Gene	Test_score	*P*_value	P_{FDR}
TRIM27	29.80	1.52E-13	4.60E-10
IRF5	27.00	6.43E-13	1.22E-09
TRIM10	91.97	7.28E-12	6.12E-09
BCL2L15	168.60	1.09E-11	7.87E-09
PUS10	280.13	2.55E-11	1.38E-08
TNPO3	196.26	2.55E-11	1.38E-08
INPP5B	19.11	1.24E-09	5.06E-07
FAM213B	18.82	2.93E-09	1.14E-06
GSDMB	17.46	6.70E-09	2.47E-06
PAM	18.02	7.33E-09	2.64E-06
MMEL1	17.32	1.04E-08	3.57E-06
UBASH3A	15.83	2.49E-08	7.87E-06
ORMDL3	15.87	5.87E-08	1.64E-05
RPS26	14.86	8.40E-08	2.18E-05
SUOX	14.02	1.10E-07	2.70E-05
AFF3	14.15	2.92E-07	6.51E-05
TSSK6	14.53	4.95E-07	1.04E-04
PGAP3	12.91	7.72E-07	1.52E-04
TYK2	12.87	8.01E-07	1.55E-04
C6orf106	11.98	2.14E-06	3.49E-04
FCRL3	11.72	3.47E-06	5.31E-04
ALDH2	11.81	3.54E-06	5.36E-04
OCIAD1	11.28	5.00E-06	7.14E-04
RNF40	10.68	6.05E-06	8.28E-04
RNASET2	10.83	8.61E-06	1.13E-03
METTL21B	10.02	1.03E-05	1.34E-03
COG6	10.85	1.06E-05	1.35E-03
HMGN4	10.18	1.79E-05	2.25E-03
HIST1H2BD	10.64	2.26E-05	2.61E-03
TRAF1	9.86	3.95E-05	4.34E-03
PPIP5K2	9.46	3.99E-05	4.34E-03
PEAK1	9.42	4.31E-05	4.63E-03
ZNF322	8.16	7.11E-05	7.08E-03
ZNF438	8.85	7.83E-05	7.65E-03
FAM167A	9.60	8.38E-05	8.03E-03
DAP	8.48	9.19E-05	8.65E-03
C5	8.66	1.23E-04	1.11E-02
PSMD5	8.36	1.30E-04	1.16E-02
SPNS1	8.27	1.57E-04	1.34E-02
GMIP	8.40	1.74E-04	1.43E-02
FAM109A	7.96	1.75E-04	1.43E-02

PSD4	6.76	2.42E-04	1.87E-02
SLPI	8.20	2.41E-04	1.87E-02
PDHB	6.86	3.03E-04	2.22E-02
TMEM50B	6.42	3.06E-04	2.23E-02
SULT1A2	6.82	3.89E-04	2.72E-02
TMEM258	6.92	4.94E-04	3.24E-02
MYH7B	5.90	5.44E-04	3.46E-02
OPRL1	6.77	5.97E-04	3.75E-02
UHRF1BP1	6.07	5.95E-04	3.75E-02
FBXO40	7.54	7.05E-04	4.27E-02
NFX1	6.43	7.26E-04	4.36E-02
FHL3	6.40	7.76E-04	4.61E-02
C6orf10	33.90	5.66E-252	8.57E-248
MUC22	8.86	7.82E-19	5.92E-15
OR2W1	8.61	7.22E-18	3.65E-14
HIST1H2BN	30.55	7.11E-14	2.69E-10
CAPZA1	29.23	2.14E-13	5.39E-10
LEMD2	27.64	3.36E-13	7.28E-10
CDSN	25.52	1.32E-12	2.22E-09
C6orf15	103.35	3.64E-12	4.24E-09
GGNBP1	68.72	3.64E-12	4.24E-09
TRIM15	68.27	3.64E-12	4.24E-09
TRIM40	69.31	3.63E-12	4.24E-09
CD28	26.55	4.06E-12	4.39E-09
SYT6	102.40	5.46E-12	5.17E-09
TRIM31	138.63	5.45E-12	5.17E-09
ZSCAN12	181.05	7.23E-12	6.12E-09
MAS1L	136.31	9.10E-12	6.89E-09
TREH	170.28	9.08E-12	6.89E-09
DCLRE1B	485.91	1.45E-11	9.97E-09
ANKRD55	125.90	1.89E-11	1.19E-08
PTPN22	512.74	1.82E-11	1.19E-08
HIPK1	377.97	2.36E-11	1.38E-08
PHTF1	762.46	2.36E-11	1.38E-08
RASGRP1	22.74	5.42E-11	2.83E-08
SCAND3	22.92	1.02E-10	5.15E-08
WDR12	22.32	1.49E-10	7.27E-08
HIST1H2BO	22.02	2.53E-10	1.20E-07
C6orf100	68.91	3.84E-10	1.76E-07
RBM17	20.42	4.21E-10	1.87E-07
IP6K3	21.24	4.42E-10	1.91E-07
ZSCAN9	20.10	4.97E-10	2.09E-07
ZGLP1	18.47	2.05E-09	8.16E-07
NPTXR	18.46	4.94E-09	1.87E-06

AP4B1	212.99	9.51E-09	3.35E-06
OR2B6	18.01	1.24E-08	4.18E-06
PADI4	16.59	1.96E-08	6.46E-06
COL5A3	16.54	2.32E-08	7.49E-06
NKAPL	17.03	3.09E-08	9.54E-06
HIST1H2AJ	16.47	3.96E-08	1.20E-05
PTPN2	16.65	4.31E-08	1.28E-05
ZNF226	16.95	4.54E-08	1.32E-05
OR11A1	-5.46	4.66E-08	1.33E-05
GPX6	-5.42	5.95E-08	1.64E-05
MIEN1	15.92	6.09E-08	1.65E-05
LEP	15.82	6.85E-08	1.82E-05
ZPBP2	16.31	8.51E-08	2.18E-05
PHF19	15.46	9.31E-08	2.35E-05
ARID5B	16.28	9.91E-08	2.46E-05
HECTD4	14.79	1.14E-07	2.75E-05
OR2B3	-5.26	1.42E-07	3.36E-05
ATP1A1	15.96	1.58E-07	3.67E-05
DDX6	14.51	2.01E-07	4.62E-05
FBXL20	14.78	2.20E-07	4.98E-05
HIST1H2AL	14.92	3.01E-07	6.60E-05
RAVER1	14.45	3.59E-07	7.77E-05
TTC34	13.50	3.86E-07	8.22E-05
STARD3	14.33	5.00E-07	1.04E-04
UBD	34.67	5.14E-07	1.05E-04
RAPGEFL1	13.70	6.42E-07	1.30E-04
ZNF433	12.17	7.11E-07	1.42E-04
TPD52	13.25	8.07E-07	1.55E-04
PDE4A	13.48	9.04E-07	1.71E-04
ZSCAN23	12.19	1.01E-06	1.89E-04
OS9	11.84	1.06E-06	1.95E-04
OR2H1	-4.87	1.10E-06	2.01E-04
CYP27B1	12.74	1.33E-06	2.37E-04
PIP4K2C	12.79	1.33E-06	2.37E-04
SLC10A4	13.25	1.35E-06	2.37E-04
BCAS2	13.31	1.54E-06	2.68E-04
GLI1	13.71	1.56E-06	2.68E-04
OLFML3	12.34	1.63E-06	2.78E-04
C16orf54	12.93	1.83E-06	3.08E-04
HIST1H4J	128.90	1.87E-06	3.11E-04
OR5V1	4.74	2.10E-06	3.46E-04
OLFM2	12.20	2.36E-06	3.81E-04
ERBB2	12.74	2.64E-06	4.21E-04
TOP2A	12.67	2.85E-06	4.49E-04

COMMD8	12.42	3.33E-06	5.15E-04
KRIT1	12.23	3.32E-06	5.15E-04
KANK2	11.93	3.64E-06	5.45E-04
TSPAN31	11.28	3.70E-06	5.50E-04
BCL7C	12.23	4.24E-06	6.22E-04
SLC39A5	12.46	4.27E-06	6.22E-04
SEZ6L2	12.06	4.49E-06	6.47E-04
METTL1	11.48	5.45E-06	7.64E-04
SLC17A2	12.11	5.42E-06	7.64E-04
IKZF3	12.61	5.59E-06	7.76E-04
HFE	11.12	6.11E-06	8.28E-04
TSFM	10.94	6.13E-06	8.28E-04
COQ10A	11.53	8.18E-06	1.10E-03
MFRP	-4.45	8.40E-06	1.12E-03
SLC1A4	11.50	9.90E-06	1.29E-03
C16orf93	10.70	1.13E-05	1.44E-03
MANEAL	10.88	1.58E-05	2.00E-03
OCIAD2	10.75	1.82E-05	2.26E-03
FAM214B	10.28	1.89E-05	2.33E-03
KCNJ5	10.73	1.94E-05	2.37E-03
PTGES3	10.33	1.97E-05	2.38E-03
KRT26	10.58	2.03E-05	2.44E-03
ADAMTS4	10.66	2.18E-05	2.60E-03
HIST1H2AI	10.58	2.20E-05	2.60E-03
SYCP1	10.61	2.21E-05	2.60E-03
LYG2	25.46	2.26E-05	2.61E-03
HIST1H2BE	10.53	2.75E-05	3.16E-03
RDH11	10.67	2.82E-05	3.21E-03
ZNF184	9.88	2.87E-05	3.24E-03
TCAP	9.94	3.13E-05	3.52E-03
PGBD1	9.85	3.19E-05	3.55E-03
AGAP2-AS1	9.76	3.24E-05	3.59E-03
SH2B3	9.78	4.17E-05	4.51E-03
SPDEF	9.68	4.72E-05	5.03E-03
HIST1H1C	9.79	4.83E-05	5.08E-03
IL12A	9.73	4.80E-05	5.08E-03
C19orf80	9.97	5.06E-05	5.21E-03
LONRF2	10.07	5.03E-05	5.21E-03
ZSWIM3	9.13	5.06E-05	5.21E-03
KCTD13	9.43	5.52E-05	5.65E-03
ARCN1	9.16	6.22E-05	6.32E-03
TBC1D10B	10.24	6.46E-05	6.52E-03
ATP10D	8.96	6.57E-05	6.59E-03
SLCO4C1	9.32	7.22E-05	7.14E-03

LAT	8.59	7.78E-05	7.65E-03
RAB14	8.71	7.88E-05	7.65E-03
LRP1	9.21	8.15E-05	7.86E-03
IL1F10	9.08	8.82E-05	8.35E-03
INO80E	7.83	8.80E-05	8.35E-03
RCC2	9.00	9.25E-05	8.65E-03
BOLA2B	7.33	9.62E-05	8.94E-03
CD86	9.22	9.77E-05	9.03E-03
LHX9	9.05	1.01E-04	9.31E-03
NPIPB11	7.75	1.04E-04	9.50E-03
FADS1	8.34	1.08E-04	9.77E-03
ATG5	8.84	1.17E-04	1.06E-02
GTPBP1	8.87	1.35E-04	1.20E-02
PNMT	9.05	1.40E-04	1.23E-02
RNF26	8.19	1.43E-04	1.25E-02
PPP1CC	8.71	1.49E-04	1.29E-02
ZBTB10	8.93	1.49E-04	1.29E-02
ZNF165	8.41	1.50E-04	1.29E-02
APOBEC3F	8.43	1.55E-04	1.33E-02
NAA25	7.97	1.59E-04	1.34E-02
DARS2	7.85	1.61E-04	1.35E-02
RDH16	7.96	1.61E-04	1.35E-02
AHCYL2	8.46	1.66E-04	1.38E-02
HSD17B6	8.41	1.72E-04	1.42E-02
MAGI3	8.31	1.79E-04	1.45E-02
MMP19	8.38	1.78E-04	1.45E-02
CBX6	8.22	1.97E-04	1.59E-02
MIB2	7.75	2.04E-04	1.63E-02
ADSL	7.76	2.10E-04	1.67E-02
TRIM59	8.43	2.14E-04	1.70E-02
NDUFA13	8.05	2.26E-04	1.78E-02
FRYL	8.14	2.33E-04	1.83E-02
BCL2L11	8.14	2.42E-04	1.87E-02
RGL3	7.52	2.58E-04	1.99E-02
PPAP2A	7.35	2.64E-04	2.02E-02
KEAP1	7.74	2.69E-04	2.05E-02
IL15	7.61	2.70E-04	2.05E-02
RPS25	7.33	2.77E-04	2.09E-02
ATP13A2	7.94	2.84E-04	2.13E-02
HEBP2	7.96	2.87E-04	2.14E-02
KIR2DL1	3.62	2.91E-04	2.16E-02
ZSCAN31	80.53	2.99E-04	2.21E-02
KRT20	7.80	3.02E-04	2.22E-02
CDK2	7.95	3.21E-04	2.32E-02

C1orf106	7.75	3.32E-04	2.39E-02
CCDC84	7.31	3.32E-04	2.39E-02
HIST1H4K	7.49	3.76E-04	2.68E-02
ELMOD1	7.29	3.81E-04	2.71E-02
DMC1	7.61	3.90E-04	2.72E-02
MSTN	8.04	3.86E-04	2.72E-02
PHLDB1	7.33	3.89E-04	2.72E-02
GRHL2	8.19	3.98E-04	2.76E-02
HIST1H2BG	8.06	4.00E-04	2.76E-02
ZNF560	7.06	4.01E-04	2.76E-02
MEF2B	7.82	4.03E-04	2.76E-02
TSPAN14	7.06	4.19E-04	2.86E-02
ECSIT	7.44	4.32E-04	2.93E-02
IGSF3	7.82	4.36E-04	2.95E-02
BOLA2	6.05	4.39E-04	2.95E-02
LCE4A	6.21	4.45E-04	2.98E-02
OR14J1	-3.51	4.48E-04	2.98E-02
ZKSCAN3	6.51	4.49E-04	2.98E-02
CCDC28A	7.33	4.57E-04	3.01E-02
CD63	7.56	4.56E-04	3.01E-02
ACAD10	7.55	5.09E-04	3.28E-02
FADS3	7.32	5.07E-04	3.28E-02
HIST1H1T	7.63	5.10E-04	3.28E-02
RIMBP3B	3.48	5.06E-04	3.28E-02
HIST1H2BM	7.33	5.30E-04	3.40E-02
SLC35E2	6.23	5.42E-04	3.46E-02
MKS1	6.59	5.70E-04	3.61E-02
YAF2	6.62	6.10E-04	3.82E-02
ANXA11	6.82	6.15E-04	3.83E-02
HIST1H4F	6.38	6.32E-04	3.92E-02
PRPF19	6.77	6.44E-04	3.98E-02
PACSIN3	7.09	6.56E-04	4.04E-02
HIST1H3I	7.40	6.63E-04	4.04E-02
TEC	6.59	6.61E-04	4.04E-02
ZSCAN5B	7.07	6.65E-04	4.04E-02
ENTHD1	7.17	7.16E-04	4.32E-02
FAM161A	6.43	7.32E-04	4.38E-02
CTF1	7.11	7.49E-04	4.46E-02
MIP	7.19	7.85E-04	4.65E-02
ABI2	6.74	7.95E-04	4.68E-02
PPM1J	6.83	8.19E-04	4.81E-02
TNFSF4	7.19	8.28E-04	4.84E-02
SLC30A7	6.77	8.37E-04	4.85E-02
TG	6.30	8.34E-04	4.85E-02

S1PR2	6.88	8.64E-04	4.99E-02

Supplementary Table 3. TWAS summary statistics in single-tissue test using whole blood eQTL from GTEx

Gene	CHR	BEST. GWAS.ID	BEST. GWAS.Z	EQTL.ID	EQTL.Z	EQTL. GWAS.Z	NSNP	TWAS.Z	TWAS.P	FDR
HLA-DRB1	6	rs477515	33.82	rs28366298	-9.93	29.98	239	-34.93	2.37E-267	1.91E-263
HLA-DQA2	6	rs477515	33.82	rs28366298	17.58	30.34	260	28.85	4.91E-183	1.98E-179
AGPAT1	6	rs477515	33.82	rs2071295	-3.51	28.37	170	-28.37	4.74E-177	1.28E-173
PRRC2A	6	rs17421624	28.90	rs2075800	6.63	27.99	283	27.99	2.09E-172	4.22E-169
PBX2	6	rs477515	33.82	rs3891175	-3.94	-17.09	170	26.33	7.96E-153	1.29E-149
NELFE	6	rs17421624	28.90	rs6907185	-4.99	-15.84	222	20.31	9.53E-92	1.28E-88
HLA-DPB2	6	rs3819721	33.27	rs2071025	9.36	-12.19	338	-18.44	5.82E-76	6.71E-73
CYP21A1P	6	rs17421624	28.90	rs1150753	-9.46	-5.33	197	16.93	2.60E-64	2.62E-61
PSORS1C3	6	rs2844509	-19.18	rs1265096	8.70	18.57	174	16.39	2.30E-60	2.06E-57
HLA-DRB6	6	rs477515	33.82	rs28366298	15.25	30.16	255	16.07	3.91E-58	3.16E-55
TAP2	6	rs477515	33.82	rs4148876	-9.36	12.08	272	-15.63	4.71E-55	3.46E-52
HLA-C	6	rs707939	27.46	rs1265087	10.07	-5.36	205	-15.61	6.27E-55	4.22E-52
HSPA1B	6	rs17421624	28.90	rs521977	4.45	-15.27	239	-14.96	1.32E-50	8.20E-48
SKIV2L	6	rs17421624	28.90	rs391165	13.27	-16.47	222	-14.69	6.97E-49	4.02E-46
TNXB	6	rs477515	33.82	rs532098	3.92	26.23	161	14.49	1.43E-47	7.70E-45
DDAH2	6	rs17421624	28.90	rs3130287	5.31	-11.59	242	-14.38	6.50E-47	3.28E-44
DDX39B	6	rs2075800	27.99	rs6929796	-10.53	11.22	277	-14.34	1.29E-46	6.13E-44
HLA-DQB1-AS1	6	rs477515	33.82	rs1063355	12.07	-9.35	220	-14.03	1.04E-44	4.67E-42
HLA-DQB2	6	rs477515	33.82	rs1063355	-18.71	-9.43	260	13.85	1.31E-43	5.57E-41
SLC44A4	6	rs17421624	28.90	rs605203	4.54	-15.63	226	-13.19	1.04E-39	4.20E-37
IFITM4P	6	rs1362126	7.95	rs1362126	7.78	7.95	38	12.86	7.23E-38	2.78E-35
FKBPL	6	rs477515	33.82	rs12153855	6.33	-14.06	163	-12.31	8.46E-35	3.11E-32
FLOT1	6	rs1265096	18.57	rs13200022	-4.94	-9.65	77	12.20	3.13E-34	1.10E-31

HLA-DQA1	6	rs477515	33.82	rs1063355	13.17	-9.35	220	-12.04	2.13E-33	7.17E-31
MICA	6	rs2075800	27.99	rs3093971	7.99	8.90	240	11.95	6.57E-33	2.12E-30
POU5F1	6	rs2844509	-19.18	rs1265098	6.95	8.75	174	11.60	4.28E-31	1.33E-28
RPL32P1	6	rs477515	33.82	rs9277935	-5.33	-10.87	329	11.38	5.43E-30	1.62E-27
RPS18	6	rs3819721	33.27	rs435945	-7.20	-6.86	469	11.27	1.80E-29	5.19E-27
PPT2	6	rs477515	33.82	rs660594	3.30	-13.19	165	-11.18	5.21E-29	1.45E-26
HLA-DRA	6	rs477515	33.82	rs2858867	3.34	-26.48	223	-10.74	6.36E-27	1.71E-24
TAPBP	6	rs3819721	33.27	rs1061783	-6.18	-10.01	496	10.37	3.24E-25	8.44E-23
HLA-B	6	rs2075800	27.99	rs6909321	9.56	-8.26	216	-10.36	3.84E-25	9.69E-23
XXbac-BPG299F13.17	6	rs2844509	-19.18	rs1966	-7.62	9.61	174	-10.07	7.80E-24	1.91E-21
NCR3	6	rs6902493	28.40	rs769178	-7.44	-4.51	302	10.02	1.26E-23	2.99E-21
RNF5	6	rs477515	33.82	rs498240	-3.80	-16.22	170	10.02	1.31E-23	3.02E-21
DAXX	6	rs3819721	33.27	rs1061783	6.01	-10.01	469	-10.01	1.41E-23	3.16E-21
BAG6	6	rs17421624	28.90	rs2534674	-4.16	-8.05	242	9.98	1.89E-23	4.13E-21
ZBTB22	6	rs3819721	33.27	rs3130100	-5.50	-9.92	486	9.92	3.31E-23	7.03E-21
MICD	6	rs1362126	7.95	rs1632953	-4.52	-5.94	37	8.83	1.09E-18	2.26E-16
HLA-DQB1	6	rs477515	33.82	rs1063355	18.99	-9.49	227	-8.66	4.81E-18	9.71E-16
MICE	6	rs1362126	7.95	rs7759272	5.67	-0.69	38	8.65	5.02E-18	9.89E-16
ZBTB12	6	rs17421624	28.90	rs3828917	13.27	-8.24	226	-8.64	5.46E-18	1.05E-15
HLA-T	6	rs1362126	7.95	rs3129063	-4.68	-4.47	37	8.50	1.91E-17	3.59E-15
HLA-DPA1	6	rs477515	33.82	rs34354079	-4.16	-8.47	321	8.03	9.88E-16	1.81E-13
GPANK1	6	rs17421624	28.90	rs35444838	5.25	-3.50	242	-8.00	1.27E-15	2.28E-13
ITPR3	6	rs2296330	13.57	rs4711336	-9.55	11.65	518	-7.95	1.85E-15	3.25E-13
HLA-DOB	6	rs477515	33.82	rs4148876	16.08	12.08	272	7.77	7.55E-15	1.30E-12
IER3	6	rs1265096	18.57	rs3130557	5.20	3.22	78	7.76	8.72E-15	1.47E-12
HCG4P5	6	rs1362126	7.95	rs2747430	-7.76	-3.64	37	7.63	2.30E-14	3.79E-12

LST1	6	rs6902493	28.40	rs4947324	-4.92	-8.27	299	7.61	2.81E-14	4.54E-12
HLA-F	6	rs1362126	7.95	rs2523405	-13.99	7.77	38	-7.48	7.47E-14	1.18E-11
HLA-J	6	rs1362126	7.95	rs2517910	-6.94	-7.38	37	7.44	9.82E-14	1.53E-11
HLA-A	6	rs1362126	7.95	rs2747430	-7.07	-3.64	37	7.43	1.10E-13	1.68E-11
MOG	6	rs1362126	7.95	rs2517910	3.99	-7.38	38	-7.38	1.59E-13	2.38E-11
HLA-DRB5	6	rs477515	33.82	rs9271055	17.58	-13.70	262	-7.29	3.10E-13	4.55E-11
HLA-DOA	6	rs477515	33.82	rs369150	-7.12	8.89	293	-7.14	9.37E-13	1.35E-10
IP6K3	6	rs2296330	13.57	rs602399	-3.95	-6.94	516	6.94	3.89E-12	5.51E-10
TYK2	19	rs2304256	-6.47	rs2304256	6.83	-6.47	376	-6.92	4.46E-12	6.21E-10
LY6G5B	6	rs17421624	28.90	rs1144709	-7.22	-7.11	242	-6.82	8.86E-12	1.21E-09
PTPN22	1	rs2476601	26.04	rs2279380	3.60	2.23	483	6.75	1.51E-11	2.03E-09
AIF1	6	rs17421624	28.90	rs6905572	-4.37	5.55	294	6.74	1.55E-11	2.05E-09
ZFP57	6	rs1362126	7.95	rs375984	18.38	-6.37	38	-6.65	2.96E-11	3.86E-09
SYNGR1	22	rs2069235	6.30	rs2069235	10.96	6.30	385	6.64	3.07E-11	3.94E-09
PGBD1	6	rs2232428	-8.63	rs1778484	3.72	-6.47	406	-6.47	1.00E-10	1.26E-08
HSD17B8	6	rs3819721	33.27	rs2855448	4.58	3.44	404	6.43	1.26E-10	1.57E-08
MICB	6	rs2075800	27.99	rs3828917	-11.41	-8.24	277	6.39	1.68E-10	2.06E-08
CUTA	6	rs453779	-17.28	rs7752202	-5.78	9.51	472	-6.37	1.86E-10	2.24E-08
GABBR1	6	rs1362126	7.95	rs375984	6.90	-6.37	38	-6.37	1.90E-10	2.26E-08
LINC00243	6	rs1265096	18.57	rs3130557	8.40	3.22	80	6.26	3.94E-10	4.61E-08
CCR6	6	rs3093024	7.62	rs3093026	-6.08	-6.27	541	6.24	4.45E-10	5.13E-08
BAK1	6	rs2296330	13.57	rs210134	15.86	-5.75	495	-5.88	4.16E-09	4.73E-07
UBASH3A	21	rs1893592	-5.73	rs1893592	12.19	-5.73	759	-5.73	9.81E-09	1.10E-06
IRF5	7	rs10488631	6.77	rs4728142	14.30	5.86	332	5.67	1.40E-08	1.55E-06
ORMDL3	17	rs2872507	5.90	rs8067378	13.07	-5.50	292	-5.65	1.64E-08	1.79E-06
VWA7	6	rs17421624	28.90	rs409558	-3.78	4.57	239	-5.62	1.90E-08	2.05E-06

IKZF3	17	rs2872507	5.90	rs907091	-6.53	-5.50	291	5.58	2.34E-08	2.49E-06
AFF3	2	rs9653442	6.95	rs2309811	5.35	4.49	456	5.56	2.67E-08	2.80E-06
GSDMB	17	rs2872507	5.90	rs8067378	14.83	-5.50	290	-5.44	5.34E-08	5.53E-06
SUOX	12	rs705699	-5.33	rs705700	-10.23	-5.29	293	5.39	7.02E-08	7.18E-06
PUS10	2	rs34695944	7.55	rs35217978	5.35	4.08	324	5.31	1.10E-07	1.11E-05
RP4-730K3.3	1	rs2476601	26.04	rs6669008	-5.22	-6.59	486	5.29	1.21E-07	1.21E-05
RP11-148O21.2	8	rs13277113	5.22	rs4840568	-6.21	4.92	601	-5.25	1.55E-07	1.53E-05
RPL23AP1	6	rs1362126	7.95	rs1632957	10.85	-3.18	38	-5.24	1.57E-07	1.53E-05
TTC34	1	rs2843401	-5.89	rs4648356	6.88	-3.76	369	-5.20	1.99E-07	1.91E-05
RP11-148O21.4	8	rs13277113	5.22	rs2736340	-5.08	5.18	602	-5.18	2.20E-07	2.09E-05
PHF19	9	rs10818482	5.62	rs11794516	-4.21	5.42	357	-5.14	2.80E-07	2.63E-05
RPS26	12	rs705699	-5.33	rs10876864	19.31	-4.79	290	-5.06	4.17E-07	3.87E-05
INPP5B	1	rs28411352	5.84	rs36084352	-15.31	-4.01	459	4.98	6.29E-07	5.77E-05
FADS2	11	rs968567	-4.95	rs968567	18.57	-4.95	414	-4.98	6.49E-07	5.89E-05
HCG4P7	6	rs1362126	7.95	rs1362126	-4.84	7.95	38	-4.90	9.46E-07	8.49E-05
IFNGR2	21	rs11702844	-5.33	rs9808753	6.71	-4.89	463	-4.89	9.98E-07	8.86E-05
RP11-973H7.4	18	rs12971201	-5.84	rs2542157	-10.13	-4.83	394	4.88	1.08E-06	9.48E-05
C6orf48	6	rs17421624	28.90	rs3749953	-8.28	5.65	239	-4.86	1.18E-06	1.02E-04
HCG9	6	rs1362126	7.95	rs1632953	-5.72	-5.94	37	4.84	1.31E-06	1.13E-04
TMEM258	11	rs968567	-4.95	rs174538	5.53	-3.66	401	-4.77	1.87E-06	1.59E-04
NEU1	6	rs17421624	28.90	rs3117575	-4.46	-3.00	226	4.74	2.11E-06	1.78E-04
FAM167A	8	rs13277113	5.22	rs4840568	12.60	4.92	624	4.68	2.88E-06	2.40E-04
HLA-DPB1	6	rs477515	33.82	rs34354079	-4.25	-8.47	328	4.65	3.35E-06	2.76E-04
RP11-973H7.1	18	rs12971201	-5.84	rs2542157	-10.08	-4.83	385	4.63	3.63E-06	2.96E-04
PGAP3	17	rs2872507	5.90	rs3816470	3.35	-5.47	318	-4.63	3.73E-06	3.01E-04
VARS2	6	rs1265096	18.57	rs3130557	-5.82	3.22	93	4.62	3.87E-06	3.09E-04

FADS1	11	rs968567	-4.95	rs102275	5.61	-3.34	421	-4.55	5.30E-06	4.20E-04
COL11A2	6	rs3819721	33.27	rs2229784	5.30	-4.53	385	-4.53	5.90E-06	4.63E-04
HCG4P3	6	rs1362126	7.95	rs3129063	5.21	-4.47	37	-4.47	7.79E-06	6.05E-04
TSPAN31	12	rs238516	-5.22	rs701008	-5.21	-4.43	341	4.43	9.60E-06	7.38E-04
METTL21B	12	rs238516	-5.22	rs10877016	14.61	-4.46	347	-4.40	1.06E-05	8.08E-04
RP11-309L24.10	7	rs10488631	6.77	rs10239340	11.71	-4.40	330	-4.40	1.10E-05	8.30E-04
FCRL3	1	rs3761959	4.38	rs3761959	12.18	4.38	537	4.38	1.20E-05	8.89E-04
TRIM10	6	rs1362126	7.95	rs1632957	-4.12	-3.18	37	4.38	1.20E-05	8.89E-04
NOTCH4	6	rs477515	33.82	rs11969759	7.42	3.85	170	4.35	1.38E-05	1.01E-03
FAM213B	1	rs2843401	-5.89	rs4445406	-9.05	-5.23	366	4.33	1.49E-05	1.08E-03
FCRL1	1	rs3761959	4.38	rs6689427	-8.28	3.72	500	-4.28	1.84E-05	1.33E-03
BLK	8	rs13277113	5.22	rs998683	-9.51	4.83	629	-4.28	1.89E-05	1.35E-03
TRAF1	9	rs10818482	5.62	rs10760130	6.60	5.33	347	4.26	2.07E-05	1.47E-03
DDR1	6	rs1265096	18.57	rs28732100	5.06	-4.25	93	-4.25	2.10E-05	1.47E-03
UBE2L3	22	rs11089637	5.00	rs4821130	8.48	4.31	286	4.25	2.12E-05	1.48E-03
MMEL1	1	rs2843401	-5.89	rs3748816	-9.37	-5.55	356	4.23	2.33E-05	1.61E-03
ZNRD1	6	rs1362126	7.95	rs3129063	-3.14	-4.47	37	4.19	2.84E-05	1.94E-03
SPNS1	16	rs7500321	4.45	rs8045689	14.21	4.17	183	4.15	3.25E-05	2.21E-03
IL12RB2	1	rs6679356	4.59	rs17129794	10.42	4.44	498	4.13	3.69E-05	2.48E-03
TMEM50B	21	rs11702844	-5.33	rs2284555	-10.23	-4.09	458	4.12	3.76E-05	2.51E-03
TRIM27	6	rs13190937	-7.31	rs9468385	5.47	-0.63	38	-4.12	3.79E-05	2.51E-03
XXbac-BPG248L24.12	6	rs2075800	27.99	rs2844623	9.58	5.11	216	4.08	4.44E-05	2.92E-03
LINC00593	15	rs8026898	8.47	rs4777182	-4.05	-4.55	533	4.03	5.67E-05	3.69E-03
CTD-2574D22.4	16	rs4787495	4.02	rs4238961	4.47	-2.72	181	-4.01	6.09E-05	3.93E-03
LINC01125	2	rs12989231	4.16	rs298913	4.22	2.71	143	3.94	8.00E-05	5.13E-03
TOP1MT	8	rs878238	3.32	rs3814772	-5.21	-2.13	369	3.91	9.35E-05	5.95E-03

GIN1	5	rs35801	-6.18	rs26262	-3.97	-3.85	318	3.90	9.59E-05	6.00E-03
RP11-235E17.6	17	rs11657606	-3.77	rs220488	-3.30	-2.48	528	3.90	9.56E-05	6.00E-03
CCT6P2	5	rs13189930	4.74	rs31929	-4.95	3.87	467	-3.87	1.10E-04	6.83E-03
FCGR2B	1	rs1801274	5.33	rs35835689	9.57	2.96	477	3.86	1.12E-04	6.90E-03
RNF40	16	rs8058578	4.67	rs8058578	6.39	4.67	209	3.86	1.13E-04	6.91E-03
ATP13A2	1	rs2240335	-5.78	rs2076600	-5.11	-3.34	344	3.81	1.39E-04	8.44E-03
DDX6	11	rs4938573	-7.76	rs2077579	-5.61	-7.60	363	3.80	1.43E-04	8.62E-03
AC093162.5	2	rs10460586	3.94	rs1053560	-8.31	3.79	446	-3.79	1.49E-04	8.91E-03
TCF7L1	2	rs10460586	3.94	rs1053560	-8.01	3.79	411	-3.78	1.58E-04	9.38E-03
TSSK6	19	rs11085264	4.24	rs998732	5.71	3.76	356	3.76	1.70E-04	1.00E-02
PSMD5	9	rs10818482	5.62	rs7037673	-4.71	-4.42	368	3.75	1.75E-04	1.02E-02
ZNF322	6	rs17539358	6.09	rs6931849	8.92	3.79	428	3.74	1.85E-04	1.07E-02
HSPA7	1	rs1801274	5.33	rs12142755	-5.96	1.73	449	-3.73	1.93E-04	1.11E-02
PLCL1	2	rs1065953	-4.25	rs1579695	11.53	-3.78	308	-3.72	1.96E-04	1.12E-02
SH2B1	16	rs7500321	4.45	rs8062405	-3.97	3.72	182	-3.72	2.00E-04	1.14E-02
FAM213A	10	rs2343306	4.47	rs12220642	-8.25	3.98	547	-3.72	2.03E-04	1.15E-02
OPRL1	20	rs4408777	3.48	rs8121509	-9.11	3.41	312	-3.71	2.10E-04	1.18E-02
HLA-G	6	rs1362126	7.95	rs9501414	-3.40	-0.43	38	3.67	2.41E-04	1.34E-02
TUFM	16	rs7500321	4.45	rs8049439	18.02	3.75	182	3.63	2.80E-04	1.55E-02
EDEM2	20	rs3746429	-3.60	rs3746429	-6.19	-3.60	345	3.62	2.97E-04	1.63E-02
ZNF831	20	rs259956	3.64	rs259964	-4.40	3.62	537	-3.62	3.00E-04	1.63E-02
EDN3	20	rs259956	3.64	rs259964	-6.46	3.62	554	-3.62	3.00E-04	1.63E-02
MYL6	12	rs705699	-5.33	rs773649	3.77	-3.61	276	-3.61	3.10E-04	1.67E-02
SMG1P5	16	rs8058578	4.67	rs9924308	-8.18	3.83	209	-3.60	3.15E-04	1.68E-02
NMI	2	rs446791	-3.66	rs4664349	5.87	2.66	448	3.59	3.32E-04	1.76E-02
PSD4	2	rs3791336	-3.95	rs3791336	16.52	-3.95	451	-3.55	3.85E-04	2.03E-02

ZSCAN26	6	rs2232428	-8.63	rs12214383	-5.35	-3.87	413	3.53	4.13E-04	2.17E-02
JOSD1	22	rs6001251	-3.63	rs5750668	-5.35	2.68	374	-3.53	4.23E-04	2.20E-02
C4B	6	rs17421624	28.90	rs1150753	11.43	-5.33	189	-3.52	4.37E-04	2.26E-02
ZNF438	10	rs867768	4.66	rs2256726	-6.25	3.38	417	-3.50	4.58E-04	2.36E-02
MAPK3	16	rs12918327	4.62	rs2005219	11.49	-3.18	227	-3.50	4.64E-04	2.37E-02
PDHB	3	rs2176082	3.81	rs4390943	-7.65	3.48	433	-3.50	4.71E-04	2.39E-02
NA	2	rs3821236	6.86	rs12693581	-7.22	-3.35	372	3.47	5.17E-04	2.59E-02
AP003774.1	11	rs660442	-3.72	rs479777	15.65	-3.66	353	-3.47	5.16E-04	2.59E-02
HOTAIRM1	7	rs6953314	-4.06	rs2960785	-16.74	3.02	420	-3.47	5.22E-04	2.60E-02
PPIP5K2	5	rs35801	-6.18	rs1011454	-15.03	-3.78	318	3.47	5.28E-04	2.61E-02
ATXN2L	16	rs7500321	4.45	rs2008514	-6.98	3.47	181	-3.47	5.30E-04	2.61E-02
RP11-122G18.11	1	rs1801274	5.33	rs4656298	-8.23	3.46	407	-3.46	5.50E-04	2.66E-02
"NABP2"	12	rs705699	-5.33	rs773643	-4.34	-2.95	258	3.46	5.49E-04	2.66E-02
ATP2A1	16	rs7500321	4.45	rs12325113	3.89	3.72	182	3.46	5.44E-04	2.66E-02
EIF3C	16	rs7500321	4.45	rs149299	5.35	1.81	201	3.43	6.06E-04	2.91E-02
CNTRL	9	rs10818482	5.62	rs2209076	6.07	-2.79	380	-3.41	6.38E-04	3.05E-02
HLA-DMA	6	rs477515	33.82	rs2854275	4.69	-11.84	286	-3.41	6.50E-04	3.08E-02
RP11-347C12.12	16	rs8058578	4.67	rs13331817	-5.43	3.29	209	-3.41	6.52E-04	3.08E-02
WAPL	10	rs3858279	-3.26	rs10887608	4.34	-3.18	440	-3.40	6.74E-04	3.16E-02
SF3A3	1	rs28411352	5.84	rs4634868	12.20	1.78	468	3.39	6.89E-04	3.22E-02
RP1-221C16.8	6	rs17539358	6.09	rs4486004	-6.94	2.62	667	-3.39	7.00E-04	3.25E-02
RP11-122G18.12	1	rs1801274	5.33	rs4656298	-7.68	3.46	396	-3.36	7.66E-04	3.53E-02
CCR3	3	rs6762266	-4.59	rs7616215	-9.62	1.76	458	-3.35	7.97E-04	3.66E-02
PAM	5	rs26258	-4.22	rs2431321	15.59	-3.35	306	-3.35	8.11E-04	3.70E-02
DAP	5	rs267949	4.91	rs5745297	10.38	-0.48	628	-3.32	8.93E-04	4.05E-02
HIST1H2BG	6	rs17539358	6.09	rs9467700	4.29	-3.22	642	-3.31	9.20E-04	4.15E-02

YAF2	12	rs7954523	-3.75	rs1669909	-4.76	-3.30	421	3.30	9.70E-04	4.35E-02
HIST1H2BF	6	rs17539358	6.09	rs2143347	4.46	2.06	652	3.30	9.80E-04	4.37E-02
FBXO40	3	rs4413346	-3.77	rs1492177	6.37	3.37	342	3.29	1.01E-03	4.46E-02
KIAA1191	5	rs1030167	-3.31	rs1030167	6.75	-3.31	314	-3.26	1.10E-03	4.80E-02
C6orf106	6	rs2814943	3.41	rs16894959	7.51	3.26	338	3.26	1.10E-03	4.80E-02
NPIPB7	16	rs7500321	4.45	rs34835	-9.58	3.26	213	-3.26	1.10E-03	4.80E-02
LMNB1	5	rs11749892	3.16	rs2271354	6.09	1.91	392	3.25	1.14E-03	4.95E-02
CMAHP	6	rs3804133	5.11	rs9295647	6.67	2.66	614	3.25	1.15E-03	4.97E-02

Supplementary Table 4. TWAS summary statistics in single-tissue test using peripheral blood eQTL from NTR

Gene	CHR	BEST. GWAS.ID	BEST. GWAS.Z	EQTL.ID	EQTL.Z	EQTL. GWAS.Z	NSNP	TWAS.Z	TWAS.P	FDR
HSPA1B	6	rs17421624	28.90	rs614549	7.02	26.22	236	27.02	8.66E-161	2.12E-157
HLA-DRB5	6	rs660895	33.82	rs9271055	23.84	-13.70	252	-25.64	5.85E-145	7.16E-142
MICA	6	rs2075800	27.99	rs2844509	-3.64	-19.18	238	21.91	2.26E-106	1.84E-103
SLC44A4	6	rs17421624	28.90	rs605203	12.78	-15.63	221	-15.68	2.22E-55	1.36E-52
BAT1	6	rs2075800	27.99	rs9267576	5.09	-12.09	273	-14.81	1.26E-49	6.17E-47
IER3	6	rs1265096	18.57	rs3130557	11.02	3.22	77	12.20	3.21E-34	1.31E-31
HLA-DOA	6	rs660895	33.82	rs447088	-4.76	11.16	280	-11.16	6.47E-29	2.26E-26
HLA-A	6	rs1362126	7.95	rs1632953	-8.60	-5.94	41	9.41	4.91E-21	1.50E-18
FLOT1	6	rs1265096	18.57	rs3130564	6.18	11.18	76	9.35	8.96E-21	2.44E-18
HLA-DQA1	6	rs660895	33.82	rs1063355	18.50	-8.04	212	-9.04	1.62E-19	3.96E-17
HLA-DOB	6	rs660895	33.82	rs4148876	12.19	12.08	261	8.69	3.76E-18	8.36E-16
HLA-F	6	rs1362126	7.95	rs2523405	-17.88	7.77	42	-7.67	1.66E-14	3.39E-12
ZNRD1	6	rs1362126	7.95	rs375984	-2.68	-6.37	41	7.51	5.75E-14	1.08E-11
DDAH2	6	rs17421624	28.90	rs389883	3.97	-14.01	239	-7.24	4.35E-13	7.60E-11
MICB	6	rs2075800	27.99	rs3828917	-9.95	-8.24	273	7.13	1.03E-12	1.68E-10
HLA-DPA1	6	rs660895	33.82	rs34354079	-5.26	-8.47	306	7.03	2.01E-12	3.07E-10
TAP2	6	rs660895	33.82	rs241440	18.19	-5.85	261	-6.39	1.62E-10	2.33E-08
HLA-G	6	rs1362126	7.95	rs375984	6.67	-6.37	42	-5.74	9.58E-09	1.30E-06
IKZF3	17	rs2872507	5.90	rs10445308	6.66	5.72	319	5.72	1.10E-08	1.42E-06
ORMDL3	17	rs2872507	5.90	rs10852936	-11.59	5.87	290	-5.56	2.74E-08	3.35E-06
GSDMB	17	rs2872507	5.90	rs3816470	11.42	-5.47	289	-5.46	4.81E-08	5.60E-06
EOMES	3	rs3806624	5.42	rs3806624	5.78	5.42	412	5.42	6.09E-08	6.77E-06
CD40	20	rs4239702	-7.55	rs9074	-4.38	-6.29	497	5.38	7.44E-08	7.92E-06

C6orf48	6	rs17421624	28.90	rs11965547	-4.35	6.35	236	-5.20	2.02E-07	1.98E-05
SYNGR1	22	rs2069235	6.30	rs2069235	6.44	6.30	392	5.20	2.00E-07	1.98E-05
FADS2	11	rs968567	-4.95	rs968567	20.25	-4.95	432	-4.95	7.38E-07	6.95E-05
MKRN2	3	rs6442323	-4.50	rs2344825	-7.51	4.01	486	-4.93	8.30E-07	7.52E-05
AFF3	2	rs9653442	6.95	rs4851274	4.25	4.29	626	4.64	3.51E-06	3.07E-04
HIST1H4F	6	rs17539358	6.09	rs17600910	4.56	4.57	605	4.57	4.81E-06	4.06E-04
ANKRD55	5	rs10065637	-8.11	rs4700266	5.04	1.41	560	4.52	6.15E-06	4.90E-04
ACAT1	11	rs4988023	-4.30	rs4754317	5.68	-4.03	369	-4.52	6.21E-06	4.90E-04
ZFP57	6	rs1362126	7.95	rs3129063	5.55	-4.47	42	-4.47	7.79E-06	5.96E-04
HIST1H2BG	6	rs17539358	6.09	rs989710	9.21	4.42	623	4.42	1.00E-05	7.42E-04
FCRL3	1	rs3761959	4.38	rs3761959	13.76	4.38	537	4.38	1.17E-05	8.42E-04
TAGAP	6	rs654690	-6.16	rs926657	-7.34	-4.13	566	4.33	1.51E-05	1.04E-03
FAM119B	12	rs238516	-5.22	rs11172349	13.40	-4.40	341	-4.32	1.53E-05	1.04E-03
CFLAR	2	rs16837131	5.02	rs13015798	5.30	-3.41	401	-4.31	1.60E-05	1.06E-03
INPP5B	1	rs28411352	5.84	rs4585948	-6.90	-4.17	498	4.23	2.33E-05	1.50E-03
TNPO3	7	rs10488631	6.77	rs6969930	5.24	-4.42	343	-4.15	3.32E-05	2.08E-03
HLA-B	6	rs2075800	27.99	rs3130564	-13.95	11.18	214	-4.11	3.90E-05	2.39E-03
ALDH2	12	rs3184504	5.12	rs16941669	5.65	-1.76	222	-4.10	4.18E-05	2.49E-03
DAP	5	rs267949	4.91	rs267949	-5.19	4.91	676	-4.08	4.60E-05	2.68E-03
GATAD2A	19	rs11085264	4.24	rs2285627	5.51	3.52	386	3.93	8.60E-05	4.89E-03
RNF41	12	rs705699	-5.33	rs773122	-3.96	-3.90	254	3.78	1.54E-04	8.56E-03
CCR3	3	rs6762266	-4.59	rs7616215	-12.86	1.76	473	-3.77	1.65E-04	8.97E-03
OCIAD1	4	rs6838613	-4.04	rs2712156	-4.50	-3.67	207	3.67	2.40E-04	1.25E-02
PPIP5K2	5	rs35801	-6.18	rs26433	-7.73	-3.87	353	3.68	2.37E-04	1.25E-02
C11orf10	11	rs968567	-4.95	rs174538	10.24	-3.66	399	-3.66	2.50E-04	1.27E-02
2-Sep	2	rs6437249	-3.36	rs11681497	11.31	-2.61	343	-3.64	2.74E-04	1.37E-02

TXK	4	rs2352593	4.49	rs4695345	4.75	-2.54	398	-3.60	3.23E-04	1.55E-02
NCOR1	17	rs11657857	-3.41	rs11650427	-4.17	-2.89	321	3.60	3.19E-04	1.55E-02
ANKRD28	3	rs11917845	3.56	rs11917845	6.36	3.56	522	3.56	3.69E-04	1.74E-02
FCGR2B	1	rs1801274	5.33	rs35835689	10.51	2.96	511	3.48	4.94E-04	2.28E-02
PLEKHA1	10	rs7097701	3.48	rs6585827	-7.92	3.45	605	-3.45	5.61E-04	2.54E-02
CCR1	3	rs6762266	-4.59	rs7616215	12.77	1.76	470	3.41	6.50E-04	2.89E-02
NPAT	11	rs4988023	-4.30	rs2356801	-4.48	2.23	380	-3.40	6.78E-04	2.96E-02
C6orf106	6	rs2814943	3.41	rs2814983	5.53	3.38	376	3.38	7.30E-04	3.13E-02
TCF19	6	rs2844509	-19.18	rs2233976	7.02	-3.37	177	-3.37	7.60E-04	3.16E-02
WEE1	11	rs2082807	-3.38	rs12422111	-3.99	1.31	367	-3.37	7.61E-04	3.16E-02
ZNF48	16	rs8058578	4.67	rs2230434	-3.63	0.09	196	-3.36	7.82E-04	3.19E-02
PAM	5	rs35801	-6.18	rs2431321	19.07	-3.35	343	-3.35	8.11E-04	3.20E-02
HIST1H2AC	6	rs17539358	6.09	rs198829	-6.29	-3.05	662	3.35	8.01E-04	3.20E-02
RETSAT	2	rs10460586	3.94	rs1044973	-11.01	3.75	452	-3.34	8.35E-04	3.24E-02
IFI30	19	rs874628	-3.57	rs11554159	5.52	-3.29	406	-3.29	9.98E-04	3.82E-02
HMGN4	6	rs17539358	6.09	rs6926629	9.46	-2.66	464	-3.27	1.07E-03	4.03E-02

Supplementary Table 5. Significant TWAS signals were validated in peripheral blood

Gene	CHR	TWAS_Z_NTR	TWAS_P_NTR	TWAS_Z_WB	TWAS_P_WB	Category
FCRL3	1	4.38	1.17E-05	4.38	1.20E-05	Known region
INPP5B	1	4.23	2.33E-05	4.98	6.29E-07	Known region
AFF3	2	4.64	3.51E-06	5.56	2.67E-08	Known region
DAP	5	-4.08	4.60E-05	-3.32	8.93E-04	Known region
PAM	5	-3.35	8.11E-04	-3.35	8.11E-04	Known region
PPIP5K2	5	3.68	2.37E-04	3.47	5.28E-04	Known region
C6orf106	6	3.38	7.30E-04	3.26	1.10E-03	Known region
HIST1H2BG	6	4.42	1.00E-05	-3.31	9.20E-04	new
GSDMB	17	-5.46	4.81E-08	-5.44	5.34E-08	Known region
IKZF3	17	5.72	1.10E-08	5.58	2.34E-08	Known region
ORMDL3	17	-5.56	2.74E-08	-5.65	1.64E-08	Known region

Supplementary Table 6. List of overlapping genes identified by TWAS and GEO mRNA profiling for RA.

Gene	CytoBand	Z score	TWAS *P*-value	TWAS FDR	t-statistics	GEO *P*-value	GEO FDR	Regulation
PTPN22	1p13.2	6.75	1.51E-11	2.03E-09	4.99	1.06E-06	8.08E-06	UP
PUS10	2p15	5.31	1.10E-07	1.11E-05	5.67	3.64E-08	4.00E-07	UP
PPIP5K2	5q21.1	3.47	5.28E-04	2.61E-02	5.05	7.88E-07	6.20E-06	UP
ZNF322	6p22.2	3.74	1.85E-04	1.07E-02	7.79	1.31E-13	5.65E-12	UP
IRF5	7q32.1	5.67	1.40E-08	1.55E-06	3.12	2.03E-03	6.44E-03	UP
PSMD5	9q33.2	3.75	1.75E-04	1.02E-02	4.83	2.24E-06	1.60E-05	UP
YAF2	12q12	3.30	9.70E-04	4.35E-02	5.95	8.07E-09	1.05E-07	UP
TMEM50B	21q22.11	4.12	3.76E-05	2.51E-03	7.35	2.21E-12	6.96E-11	UP
PGBD1	6p22.1	-6.47	1.00E-10	1.26E-08	-2.15	3.28E-02	6.95E-02	DOWN
PHF19	9q33.2	-5.14	2.80E-07	2.63E-05	-2.32	2.12E-02	4.84E-02	DOWN
PGAP3	17q12	-4.63	3.73E-06	3.01E-04	-3.71	2.50E-04	1.04E-03	DOWN
GSDMB	17q21.1	-5.44	5.34E-08	5.53E-06	-2.72	6.93E-03	1.85E-02	DOWN
ORMDL3	17q21.1	-5.65	1.64E-08	1.79E-06	-2.89	4.16E-03	1.20E-02	DOWN
TYK2	19p13.2	-6.92	4.46E-12	6.21E-10	-2.72	6.96E-03	1.86E-02	DOWN
UBASH3A	21q22.3	-5.73	9.81E-09	1.10E-06	-6.49	3.87E-10	7.08E-09	DOWN

Supplementary Table 7. Gene-set analysis by TWAS-based enrichment analysis

GeneSet	Estimate	SE	T	N_Mem_ Avail	N_Mem	P	P.CORR
KEGG_AUTOIMMUNE_THYROID_DISEASE	1.163	0.229	5.071	18	52	1.98E-07	1.46E-05
KEGG_ALLOGRAFT_REJECTION	1.163	0.229	5.071	18	37	1.98E-07	1.46E-05
KEGG_GRAFT_VERSUS_HOST_DISEASE	1.049	0.218	4.820	20	41	7.18E-07	3.54E-05
KEGG_TYPE_I_DIABETES_MELLITUS	0.997	0.225	4.429	19	43	4.72E-06	1.75E-04
KEGG_VIRAL_MYOCARDITIS	0.732	0.177	4.133	31	70	1.79E-05	5.29E-04
KEGG_ANTIGEN_PROCESSING_AND_PRESENTATION	0.655	0.177	3.702	35	88	1.07E-04	2.64E-03
KEGG_INTESTINAL_IMMUNE_NETWORK_FOR_IGA_PRODUCTION	0.859	0.236	3.643	19	48	1.35E-04	2.85E-03
KEGG_ASTHMA	0.919	0.275	3.340	14	30	4.19E-04	7.75E-03
KEGG_CELL_ADHESION_MOLECULES_CAMS	0.523	0.159	3.293	44	133	4.96E-04	8.16E-03
BIOCARTA_NKT_PATHWAY	1.190	0.363	3.281	10	26	5.17E-04	1.40E-02

Supplementary Table 8. Gene-set analysis of significant genes identified by MAGMA

GeneSet	NGENES	BETA	BETA_STD	SE	P	FDR
KEGG_AUTOIMMUNE_THYROID_DISEASE	48	1.457	0.075	0.178	1.53E-16	2.85E-14
KEGG_ALLOGRAFT_REJECTION	33	1.529	0.065	0.194	1.51E-15	1.40E-13
KEGG_GRAFT_VERSUS_HOST_DISEASE	34	1.271	0.055	0.192	2.07E-11	1.28E-09
KEGG_TYPE_I_DIABETES_MELLITUS	40	0.975	0.046	0.165	1.55E-09	7.23E-08
BIOCARTA_TH1TH2_PATHWAY	19	1.426	0.046	0.234	5.86E-10	1.71E-07
KEGG_INTESTINAL_IMMUNE_NETWORK_FOR_IGA_PRODUCTION	44	0.861	0.042	0.159	3.36E-08	1.25E-06
KEGG_CELL_ADHESION_MOLECULES_CAMS	123	0.474	0.039	0.092	1.22E-07	3.80E-06
BIOCARTA_CTLA4_PATHWAY	19	1.384	0.045	0.263	6.96E-08	1.02E-05
REACTOME_INTERLEUKIN_2_FAMILY_SIGNALING	39	1.016	0.047	0.185	2.09E-08	3.37E-05
KEGG_VIRAL_MYOCARDITIS	65	0.564	0.034	0.128	5.55E-06	1.47E-04
KEGG_CYTOKINE_CYTOKINE_RECEPTOR_INTERACTION	249	0.317	0.037	0.073	7.02E-06	1.63E-04
REACTOME_INTERFERON_GAMMA_SIGNALING	86	0.566	0.039	0.112	2.27E-07	1.84E-04
KEGG_SYSTEMIC_LUPUS_ERYTHEMATOSUS	122	0.482	0.039	0.113	1.05E-05	2.17E-04
KEGG_JAK_STAT_SIGNALING_PATHWAY	147	0.384	0.034	0.091	1.17E-05	2.18E-04
KEGG_LEISHMANIA_INFECTION	65	0.499	0.030	0.124	3.08E-05	5.21E-04
KEGG_NATURAL_KILLER_CELL_MEDIATED_CYTOTOXICITY	128	0.374	0.031	0.095	4.07E-05	6.30E-04
KEGG_ANTIGEN_PROCESSING_AND_PRESENTATION	78	0.471	0.031	0.123	6.76E-05	9.67E-04
KEGG_ASTHMA	26	0.702	0.027	0.185	7.32E-05	9.72E-04
BIOCARTA_ASBCELL_PATHWAY	11	1.414	0.035	0.343	1.90E-05	1.39E-03
KEGG_T_CELL_RECEPTOR_SIGNALING_PATHWAY	105	0.345	0.026	0.096	1.68E-04	2.08E-03
REACTOME_CYTOKINE_SIGNALING_IN_IMMUNE_SYSTEM	679	0.172	0.033	0.039	5.77E-06	3.10E-03
REACTOME_COSTIMULATION_BY_THE_CD28_FAMILY	65	0.551	0.033	0.129	9.89E-06	3.94E-03
REACTOME_PD_1_SIGNALING	20	1.006	0.033	0.238	1.22E-05	3.94E-03
REACTOME_ADAPTIVE_IMMUNE_SYSTEM	720	0.152	0.030	0.038	2.67E-05	7.18E-03

REACTOME_REGULATION_OF_IFNG_SIGNALING	14	1.021	0.028	0.257	3.52E-05	8.11E-03
REACTOME_INTERLEUKIN_2_SIGNALING	11	1.369	0.034	0.351	4.72E-05	9.54E-03
REACTOME_RAP1_SIGNALLING	16	1.039	0.031	0.270	6.05E-05	1.09E-02
REACTOME_INTERLEUKIN_3_INTERLEUKIN_5_AND_GM_CSF_SIGNALING	44	0.622	0.031	0.165	8.25E-05	1.21E-02
REACTOME_INTERFERON_SIGNALING	194	0.272	0.028	0.073	9.40E-05	1.26E-02
KEGG_LONG_TERM_POTENTIATION	66	0.366	0.022	0.120	1.18E-03	1.37E-02
BIOCARTA_IL2_PATHWAY	20	0.824	0.027	0.236	2.43E-04	1.42E-02
BIOCARTA_IL2RB_PATHWAY	36	0.529	0.024	0.155	3.25E-04	1.58E-02
BIOCARTA_MHC_PATHWAY	11	0.916	0.023	0.276	4.56E-04	1.67E-02
BIOCARTA_FAS_PATHWAY	30	0.549	0.022	0.165	4.41E-04	1.67E-02
BIOCARTA_BLYMPHOCYTE_PATHWAY	11	0.969	0.024	0.299	5.84E-04	1.89E-02
REACTOME_TERMINATION_OF_O_GLYCAN_BIOSYNTHESIS	23	0.739	0.026	0.206	1.67E-04	2.08E-02
REACTOME_E3_UBIQUITIN_LIGASES_UBIQUITINATE_TARGET_PROTEINS	57	0.482	0.027	0.139	2.62E-04	2.83E-02
KEGG_VASCULAR_SMOOTH_MUSCLE_CONTRACTION	112	0.261	0.020	0.094	2.84E-03	3.11E-02
REACTOME_PROTEIN_UBIQUITINATION	75	0.409	0.026	0.120	3.29E-04	3.11E-02
REACTOME_INTERFERON_ALPHA_BETA_SIGNALING	70	0.429	0.027	0.127	3.47E-04	3.11E-02
REACTOME_INTERLEUKIN_RECEPTOR_SHC_SIGNALING	23	0.810	0.029	0.238	3.42E-04	3.11E-02
REACTOME_SYNTHESIS_OF_IP2_IP_AND_INS_IN_THE_CYTOSOL	13	0.768	0.021	0.231	4.51E-04	3.64E-02
REACTOME_FCERI_MEDIATED_CA_2_MOBILIZATION	30	0.614	0.025	0.187	5.04E-04	3.88E-02
REACTOME_INNATE_IMMUNE_SYSTEM	1003	0.106	0.024	0.032	5.37E-04	3.94E-02
REACTOME_SIGNALING_BY_INTERLEUKINS	436	0.156	0.024	0.048	6.50E-04	4.57E-02
