# Supplementary material for: Novel insight into the aetiology of rheumatoid arthritis gained by a cross-tissue transcriptome-wide association study
Source: RMD Open. 2022 Sep 8;8(2):e002529. doi: 10.1136/rmdopen-2022-002529 (PMC9462377; doi:10.1136/rmdopen-2022-002529)
Supplement: Supplementary data [file rmdopen-2022-002529supp001.pdf]

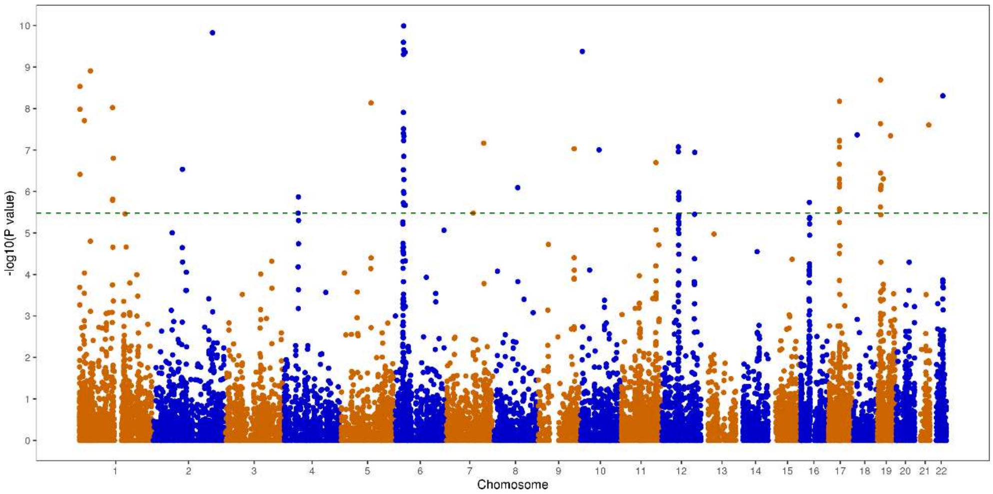

Supplementary Figure 1. Manhattan plot of the cross-tissue transcriptome-wide association results for RA. Nighty-six genes was specifically associated with the risk of RA. The y-axis represents *P*-value in –log(10) scale. A significance threshold of *P* = 2.89×10-6 after Bonferroni-correction was used.

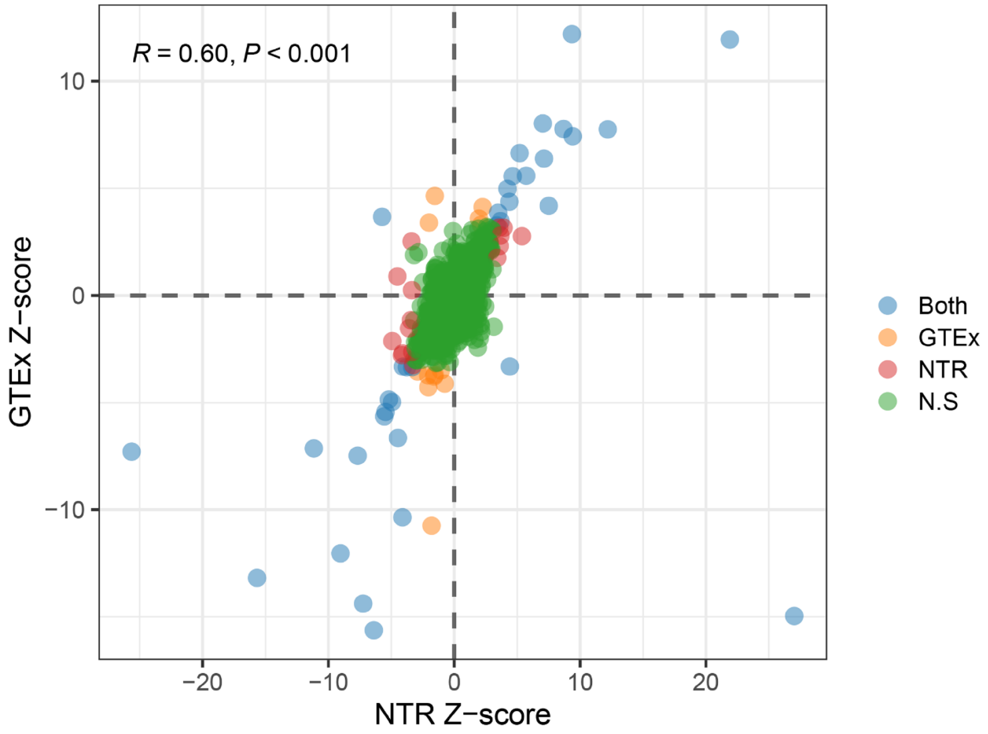

Supplementary Figure 2. Correlation plot shows the correlation of the effect sizes of TWAS between GTEx and NTR.

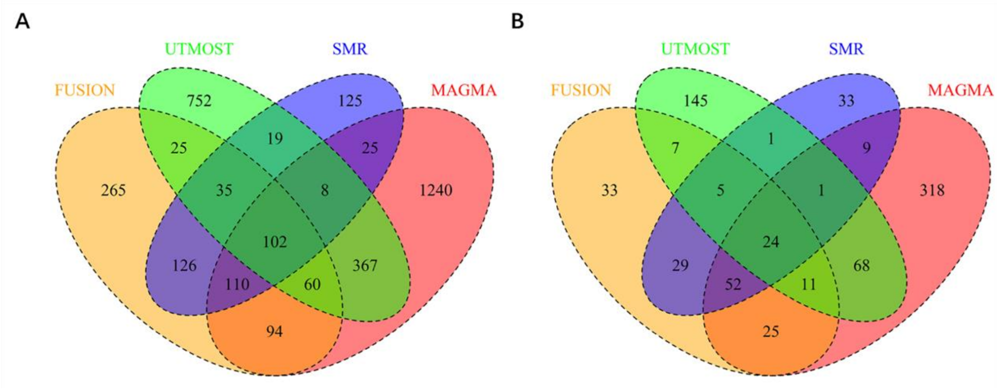

Supplementary Figure 3. Venn plot reveals the overlap of the significant genes identified by four different methods with $P < 0.05$ (A) and FDR < 0.05 (B).

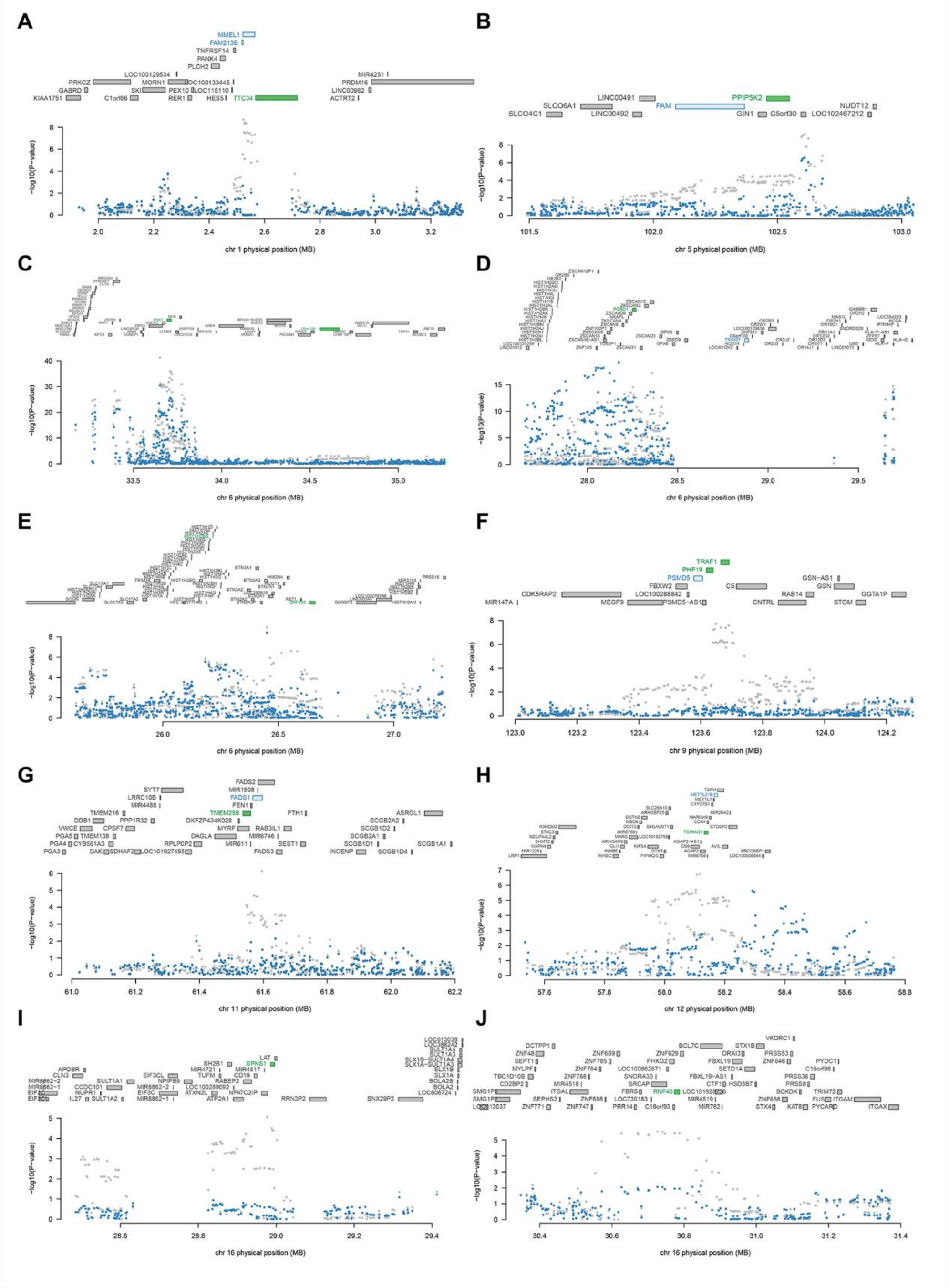

Supplementary Figure 4. Regional association of transcriptome-wide significant genes. **A:** Chromosome 1p36.32 regional association plot. **B:** Chromosome 5q21.1 regional association plot. **C:** Chromosome 6p21.31 regional association plot. **D:** Chromosome 6p22.1 regional association plot. **E:** Chromosome 6p22.2 regional association plot. **F:** Chromosome 9q33.2 regional association plot. **G:** Chromosome 11q12.2 regional association plot. **H:** Chromosome 12q14.1 regional association plot. **I:** Chromosome 16p11.2 regional association plot. **J:** Chromosome 16p11.2 regional association plot.
